# Stress Conditions Modulate the Chromatin Interactions Network in *Arabidopsis*

**DOI:** 10.3389/fgene.2021.799805

**Published:** 2022-01-05

**Authors:** Vikash Kumar Yadav, Swadha Singh, Amrita Yadav, Neha Agarwal, Babita Singh, Siddhi Kashinath Jalmi, Vrijesh Kumar Yadav, Vipin Kumar Tiwari, Verandra Kumar, Raghvendra Singh, Samir Vishwanath Sawant

**Affiliations:** ^1^ CSIR-National Botanical Research Institute, Lucknow, India; ^2^ Academy of Scientific and Innovative Research (AcSIR), Ghaziabad, India; ^3^ School of Natural Sciences, University of California, Merced, Merced, CA, United States; ^4^ Department of Botany, Goa University, Taleigão, India; ^5^ Department of Botany, Manyawar Kanshiram Government Degree College, Aligarh, India; ^6^ CSIR-Centre for Cellular & Molecular Biology, Hyderabad, India

**Keywords:** stress, chromatin-chromatin interactions, genome organization, epigenetic state, Hi-C, heterochromatin, QTL

## Abstract

Stresses have been known to cause various responses like cellular physiology, gene regulation, and genome remodeling in the organism to cope and survive. Here, we assessed the impact of stress conditions on the chromatin-interactome network of *Arabidopsis thaliana*. We identified thousands of chromatin interactions in native as well as in salicylic acid treatment and high temperature conditions in a genome-wide fashion. Our analysis revealed the definite pattern of chromatin interactions and stress conditions could modulate the dynamics of chromatin interactions. We found the heterochromatic region of the genome actively involved in the chromatin interactions. We further observed that the establishment or loss of interactions in response to stress does not result in the global change in the expression profile of interacting genes; however, interacting regions (genes) containing motifs for known TFs showed either lower expression or no difference than non-interacting genes. The present study also revealed that interactions preferred among the same epigenetic state (ES) suggest interactions clustered the same ES together in the 3D space of the nucleus. Our analysis showed that stress conditions affect the dynamics of chromatin interactions among the chromatin loci and these interaction networks govern the folding principle of chromatin by bringing together similar epigenetic marks.

## Introduction

The haploid *A. thaliana* genome contains approximately 125 million base pairs of DNA packaged into five chromosomes (Kaul et al., 2000). That makes a total 250 million base pairs of DNA in a single diploid cell of *A. thaliana*, which spans a total length of ∼8.5 cm. This stretch of DNA is approximately 16,000 times larger than the diameter of *A. thaliana* nucleus (Dittmer et al., 2007). Thus, the DNA packaging in the nucleus is accomplished in a very organized way which prevents the DNA from becoming an unmanageable tangle. Interestingly, after being packaged so compactly it manages in such a way that their distal regulatory elements remain accessible to their target gene for their regulation. Chromatin-chromatin interaction has been identified as an important mechanism for such regulation (Li et al., 2012; Sun et al., 2020; Yadav et al., 2021). A distal element can specifically interact with its target gene situated on the same or different chromosome by looping (Dean, 2011). Loop formation is thus an integral part of chromatin organization which facilitates interactions between distal genomic elements (Deng et al., 2012; Sandhu et al., 2012).

Several high throughput approaches such as Formaldehyde-Assisted Isolation of Regulatory Elements (FAIR) (Simon et al., 2012), Chromatin Immunoprecipitation Sequencing (ChIP-Seq) (Furey, 2012), and DNaseI-Seq (Song and Crawford, 2010) are available as standard experimental methods for the identification of regulatory elements. However, their primary limitations are that they cannot determine the precise association of distal regulatory elements with target genes and spatial conformation required for their optimal activity. Previously, cytogenetic techniques and microscopic observation have been used to study chromosomal organization but over the last 20 years, our knowledge about chromosomal architecture enhances with the advancement of high-resolution techniques. There are several techniques which have been used widely to identify local and global chromatin interactome network, for example, chromosome conformation capture (3C) (Dekker et al., 2002), circular chromosome conformation capture (4C) (Zhao et al., 2006), chromosome conformation capture carbon copy (5C) (Dostie et al., 2006), high-throughput chromosome conformation capture (Hi-C) (Lieberman-Aiden et al., 2009), DNase Hi-C (Ma et al., 2018), Capture Hi-C (Eijsbouts et al., 2019), INT-Hi-C (Yadav et al., 2021), and chromatin interaction analysis using paired-end tag (ChIA-PET) (Fullwood and Ruan, 2009).

Three-dimensional chromatin organization is necessary for many biological processes including transcriptional regulation, replication, and repair (Ling et al., 2006; Zhao et al., 2006). A series of publications on *A. thaliana* showed interest of the plant community in chromatin organization and its function (Moissiard et al., 2012; Grob et al., 2013; Grob et al., 2014; Feng et al., 2014; Sequeira-Mendes et al., 2014; Wang et al., 2015, 2016; Zhu et al., 2015; Liu et al., 2016; Bi et al., 2017; Zhang et al., 2019; Yadav et al., 2021). Chromatin architecture of wild-type and mutant (atmorc6-1) *A. thaliana* showed a similar pattern although the interaction frequency varies among different chromatin regions in wild-type and mutant (Moissiard et al., 2012). Further, it is reported that chromosomes interact with each other via pericentromeric and heterochromatic regions (Feng et al., 2014). There exists a strong correlation between chromosomal architecture and epigenetic landscape (Grob et al., 2014; Yadav et al., 2021). The importance of chromatin looping was identified in the b1 locus of maize which is required for both paramutation and its high expression (Louwers et al., 2009a). Similarly, distal regulatory element is required for gene activation of FLC locus in *A. thaliana* (Crevillén et al., 2012). Thus, it is interesting and important to identify these distal regulatory elements in plants and their regulation. Thus, global mapping of chromatin interactions in *A. thaliana* is likely to uncover the genome architecture and its regulation.

Plants being sessile organisms always faced various stress conditions (both biotic and abiotic), however, very few efforts have been made to understand how stress conditions influence the chromatin interactions (Sun et al., 2020; Li et al., 2021; Liang et al., 2021). Thus, our present study assesses the impact of biotic and abiotic stresses on plant chromatin interaction networks. We used Hi-C to identify chromatin interactions and the impact of stress conditions on chromatin interactome. Our study revealed thousands of chromatin interactions in native condition (NC), heat treatment (HT, abiotic stress), and salicylic acid (SA, mimic biotic stress) treated *A. thaliana* and the impact of stresses on these interaction dynamics. We also investigate the correlation between epigenetic state (ES), chromatin interaction network, and gene expression. Our study will help to understand how the stress conditions affect the chromatin organization in plants which may directly or indirectly affect the genome regulation, and hence the organism response to the external environment.

## Materials and Methods

### Plant Material

Seeds of *A. thaliana* (Columbia-0 ecotype) (ABRC; https://abrc.osu.edu/) were germinated and grown for 6 weeks on solrite under the long-day conditions (22°C, 16-h light and 8-h dark), after seeds have been stratified on soil (solrite) supplemented with water at 4°C for 4 days. For SA (Sigma) treatment 2 mM SA was sprayed on the aerial part of the plant material 2 days before nuclei were harvested. Plants grown as mentioned were exposed to 40°C for 1 h for the high-temperature treatment. The aerial tissue of native condition (NC) and treated plants (SA and HT) were used for preparing Hi-C and RNA-Seq libraries.

### Fixation of Plant Tissue and Nuclei Preparation

The aerial tissue of 6-week-old NC, SA, and HT treated plants were cross-linked for 1 h separately by adding 37% formaldehyde to a final concentration of 1% in extraction buffer (2 M Hexylene glycol, 20 mM PIPES, pH 7.0, 10 mM MgCl_2_, 5 mM ß-mercaptoethenol). After cross-linking the remaining formaldehyde was sequestered by adding 1/16 volume of 2 M ice-cold glycine for 10 min. The remaining solution was decanted, and tissue was rinsed three times with ice-cold milli-Q (MQ). Eventually, tissues were dried using paper towels and frozen in liquid N_2_. Nuclei were isolated from the cross-linked samples as described (Bowler et al., 2004; Louwers et al., 2009b). The quality of nuclei was checked using fluorescent microscopy by DAPI staining and DNA quality and quantity were estimated using agarose gel electrophoresis and Quant-iT assay.

### Hi-C Libraries Preparation and Sequencing

A total of 6 Hi-C libraries, 2 for NC, 2 for SA and HT treated samples were prepared according to Lieberman-Aiden et al. (2009) and van Berkum et al. (2010) with some modifications adapted for plant samples as described in Louwers et al. (2009b). See Supplementary File S4 for a detailed experimental procedure. Amplified Hi-C libraries were sequenced on a HiSeq2500 sequencer obtaining paired-end (100 × 2 bp) reads.

### RNA-Seq and Data Analysis

The 6 RNA libraries for three conditions, two for native condition Col-0 (NC), two for HT and two for SA treated samples were prepared. Briefly, total RNA was isolated using Spectrum™ Plant Total RNA Kit (Sigma) and libraries were prepared with the Illumina standard protocol. RNA-seq reads were aligned to *A. thaliana* reference genome (TAIR10) using TopHat with default parameters (Trapnell et al., 2009). Normalized FPKM (fragments per kilobase per million mapped reads) counts for each gene were calculated using Cufflink (Trapnell et al., 2012). The differentially expressed genes (up- and down-regulated gene) were determined by log2 fold change between untreated (NC) and treated (HT and SA) samples.

### Mapping and Filtering Uninformative Reads

For Hi-C analysis, reads were filtered based on the quality score using FASTX-Toolkit (http://hannonlab.cshl.edu/fastx_toolkit/index.html). Each end of paired reads was aligned separately to the *A. thaliana* reference genome (TAIR 10) using Bowtie v1.1.0 (Swarbreck et al., 2007; Langmead et al., 2009). Mapped reads were used for further downstream analysis. Read pairs that were not representative of true interactions like continuous genomic fragments, self-ligation, or re-ligation products were omitted using HOMER command line–removePEbg–removeSelfLigation. We had considered only those reads in which one or both of the paired-end reads have HindIII restriction site within the fragment length estimated from 3′ end of the reads (Heinz et al., 2010).

### Normalizing Hi-C Data

Hi-C data was normalized to avoid the biasness due to its mapping ability, variable number of restriction sites in a region, or technical artifacts (inaccessibility of restriction enzyme to the DNA) and linear distance between interacting regions. The expected number of reads in any given genomic region is calculated based on the number of reads in all other regions of the genome. The expected number of reads between any two regions depends on both the linear distance and sequencing depth of the library. The expected number of reads was calculated with the following equation (Heinz et al., 2010).

$$\mathrm{eij}=f\left(i-j\right)\frac{\left(n^{*}i\right)\left(n^{*}j\right)}{N^{*}}$$

where f is the expected number of reads as a function of distance, N^*^ is the total number of reads, and n^*^ is the estimated number of interacting reads at each region i and j. HOMER used simple hill climbing optimization to calculate inferred total reads through the difference between the observed and the expected number of reads.

### Generation of Interaction Matrices

The interaction matrix is the simplified way to represent the Hi-C data where interaction frequencies between any two loci can be visualized. To create the contact matrix, the genome was divided into 200 kb bin size (Moissiard et al., 2012). The interaction matrix was generated based on the frequency of interacting reads between the two bins. Contact matrix corresponds to the number of interacting reads between locus i and j. The row and column correspond to coordinates of genomic regions and the corresponding value provides interaction information between each locus. We had generated normalized and correlation interactions matrices. Interaction matrices were normalized assuming that each bin has an equal chance of interaction with all other bins in the genome and computed by ratios between the total observed and the expected number of reads in a given bin size. The interaction matrix reveals which parts of chromosomes are positioned close together or apart from each other in the nucleus. A correlation matrix is based on the Pearson correlation coefficient and considers how each bin interacts with all other bins.

### Data Visualization

To visualize high-resolution interaction data, we generated heat maps with MeV (v4.9) using interaction matrices (Saeed et al., 2003). Circos (v0.69) was used to visualize significant *cis* and *trans* interaction networks (Krzywinski et al., 2009).

### Identification of Significant Interaction and Annotation

Significant interactions were identified based on the premise behind the enrichment of observed interacting reads over the expected. It searches the genome for a pair of loci that have more interacting reads (observed) than would be expected by chance. For two potentially interacting loci, HOMER model their expected read count using the cumulative binomial distribution, where it calculates the expected read count possibly mapped between the genomic loci and the number of observed read count between the genomic loci. We had identified the significant interactions at a resolution of 1 Kb with *p*-value cut-off 0.05 at default parameter. To extend this, we annotate these significant interactions to explore what these coordinates represent in *A. thaliana* genome using TAIR10 annotation.

### Expression Profile of Interacting Genes

To check the effect of interactions on the expression profile of interacting genes, we analyzed the expression profile of interacting genes with the control sets of genes. For control, we choose an equal number of random genes that are identified as non-interacting in our study. The expression of both interacting and non-interacting sets was extracted from the RNA-seq data of the respective condition (Supplementary File S5).

### Motifs Identification

For the identification of conserved motifs, we considered only those interacting sequences in which at least one partner was a protein-coding gene. Sequences corresponding to these regions in all three conditions (NC, HT, and SA) were subjected to motif identification through MEME (http://meme.nbcr.net/meme/tools/meme). One kb region of interacting sequences was extracted from TAIR10. Total 1485, 1028 and 1196 non-redundant sequences of HT, SA, and NC were subjected to motif prediction. MEME, v4.11.2, was deployed using ZOOPS model, with motif width 6–10 bases, Evalue 0.001, and maximum numbers of motifs to return were 10 (Bailey et al., 2009). Predicted motifs were further annotated with the help of the STAMPS tool using the AGRIS database (The *Arabidopsis* Gene Regulatory Information Server, http://*Arabidopsis*.med.ohio-state.edu/) and identified the binding site of known TFs in the interacting regions (Mahony and Benos, 2007; Yilmaz et al., 2011).

### Association of Interacting Regions with Epigenetic States

To identify whether the captured interacting regions have any preferential distribution in previously reported 9 ES (Sequeira-Mendes et al., 2014), the coordinates of interacting regions were mapped onto the coordinates of 9 different ES of *A. thaliana* to identify the ES in the interacting regions. One region may fall into more than one ES as these predefined ES have overlapping regions.

### GWAS Enrichment

We mapped the publicly available GWAS hit (Atwell et al., 2010) to the interacting and non-interacting region (control). Background frequency was calculated as total unique SNP per bp of the genome.

## Results

### Stress Condition Modulate Chromatin Interactome

**FIGURE 1:**
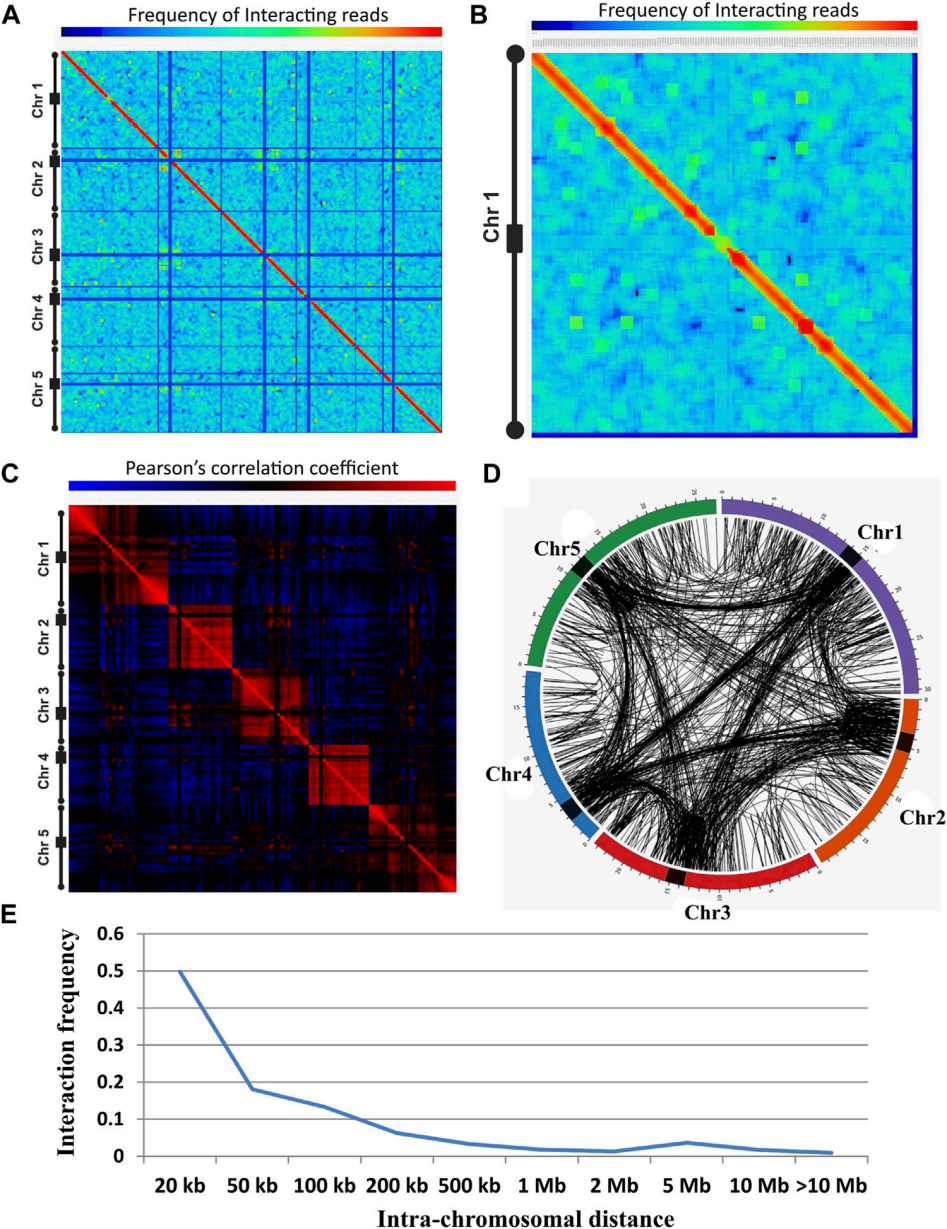
Chromatin interactions landscape in *A. thaliana*. **(A)** Genome-wide normalized interaction matrix at 200 kb resolution for *Arabidopsis* genome (NC), showing intense red diagonal representing the enrichment of interacting reads in close proximity. Blue line represents reads that are less enriched in the centromeric and telomeric region of the genome. The color bar ranging from blue to red represents the lower to higher enrichment of interacting reads. The five different chromosomes are indicated with a black line, in which a rectangular box represents the pericentromeric and circles represent the telomeric region of the chromosome. **(B)** Normalized interaction matrix for chromosome 1 showing several spots of enriched interacting reads in the genome indicating the presence of long-distance chromatin-chromatin interactions. **(C)** Genome-wide correlation interaction matrix representing the correlation among the interacting region of HT library. The red color represents the positive correlation and the blue color represents the negative correlation between the two regions. Correlation matrix suggests two distinct regions of the genome showing positively correlated interacting regions and negatively correlated non-interacting regions. **(D)** Circos representing the genome-wide identified significant interaction of NC library. Interactions were represented in the chromosomes through the black line connecting the two points (interacting regions). Spans that link the regions within the chromosome represent *cis* interactions while spans that link the regions between the chromosomes represent *trans* interactions. The outermost colored circle is the graphical representation of *Arabidopsis* chromosomes and the black rectangular box on it represents the centromeric region of each chromosome. Chromosome numbers are indicated after the chromosome (Chr) abbreviation. **(E)** Plot showing the relationship of interactions frequency with the linear physical distance along the chromosome for NC library. Intra-chromosomal interaction frequency decreases with increasing linear distance on the chromosomes.

The present study aims at understanding the dynamics of chromatin interaction during stress conditions, thus HT representing abiotic stress and SA mimic biotic stress was selected to capture chromatin interactions. We captured the chromatin interactions in NC and in HT and SA treated *A. thaliana* (Col-0) using Hi-C. A total of ∼262 million paired reads were obtained, and of these ∼87 million for NC (43.5 and 43.4 million for biological replicates 1 and 2), ∼81 million for HT (39.7 and 41.6 million for biological replicates 1 and 2), and ∼94 million for SA (55.8 and 38.6 million for biological replicates 1 and 2). To increase the depth of data we combined the biological replicates in the subsequent analysis. Heat map at a resolution of 200 kb effectively shows the interactions within chromosome arms, between arms, and between the chromosomes, and exhibits distinct substructure in the form of an intense diagonal (Figure 1A). The intense diagonal indicates that the majorities of interacting reads are a short distance within the 200 kb (Figure 1A; Supplementary Figure S1). Further, we observed fewer interacting reads between the centromere and the rest of the genome in NC, HT, and SA conditions. To have a closer inspection of chromosomal regions that interact with each other, we plotted a normalized interaction matrix for individual chromosomes. It showed many blocks enriched in interacting reads over a long distance within and between the arms of the same chromosome (Figure 1B; Supplementary Figure S2). These enriched blocks are found across all chromosomes and in all conditions with variations in positioning and intensity of blocks at different conditions (Supplementary Figure S2).

We plotted the correlation matrix to identify how each locus interacts with all other loci on a chromosome. The correlation heat map showed two distinct types of compartments within a chromosome; one compartment showed correlated regions (red) and the other represented non-correlated regions (blue) (Figure 1C; Supplementary Figure S3). This correlation plot indicates there are a preferential enrichment and depletion of interacting reads among many chromatin loci. Strong correlation was found along the diagonal similar to the interaction matrices showing enrichment of reads in the neighborhood. The correlation matrix showed less correlation among the centromere and with the rest of the genome indicating that interacting reads between the centromere and another part of the genome are scanty (Figure 1C; Supplementary Figure S3).

We identified potential statistically significant (*p*-value ≤ 0.05) chromatin interactions at a resolution of 1 kb. Our analysis revealed a total of 3635, 5320, and 3309 statistically significant interactions for NC, HT, and SA libraries, respectively (Supplementary Table S1). Based on the positioning of these interacting loci on a chromosome, these interactions were designated as *cis* (intra-chromosomal, both interacting loci found on the same chromosome including interactions between homologous chromosomes) or *trans* (inter-chromosomal, both interacting loci located on a different chromosome) interactions. In NC out of 3635 total significant interactions, 2698 were *cis* interactions and 937 were *trans* interactions. For HT and SA treated, out of a total 5320 and 3309 significant interactions 3731 and 2288 were assigned as *cis*, and 1589 and 1021 as *trans,* respectively (Supplementary Table S1 and Supplementary File S3). For the *cis* interactions, the median distances between interactions were 20,118, 24,392, and 25,770 bps, for NC, HT, and SA conditions, respectively, indicating the captured interactions were indeed both short and long range.

Further, the Circos plot of intra- and inter-chromosomal contacts indicates that the interactions were enriched in the centromeric regions for all five chromosomes (Figure 1D; Supplementary Figure S4). In general, the visual pattern of the chromosomal interaction profile of NC did not change on HT and SA treatment although the frequency of interactions between the chromatin loci may vary due to stress conditions (Figure 1D; Supplementary Figure S4). We found that telomere preferentially interacts with the other telomere and centromere of the same or different chromosomes (Figure 1D; Supplementary Figure S4).

We also identified that the number of intra-chromosomal contact probability decreases as a function of genomic distance in the base pair along the linear chromosome (Dekker et al., 2002; Lieberman-Aiden et al., 2009), and it was similar in all three conditions (Figure 1E; Supplementary Figure S4). Next, we identified the common interaction through overlapping interacting regions in all three conditions. A total of 2531, 2413, and 2422 interactions were shared between NC and HT, between NC and SA, and between HT and SA, respectively. On comparison of all three conditions, 2059 interactions were common, 750, 2426, and 533 interactions were exclusive to NC, HT, and SA conditions, respectively (Supplementary Figure S5). The result indicates that the stress conditions affect the dynamics of chromatin interactions.

### Impact of Chromatin Interactome on Gene Expression

**FIGURE 2:**
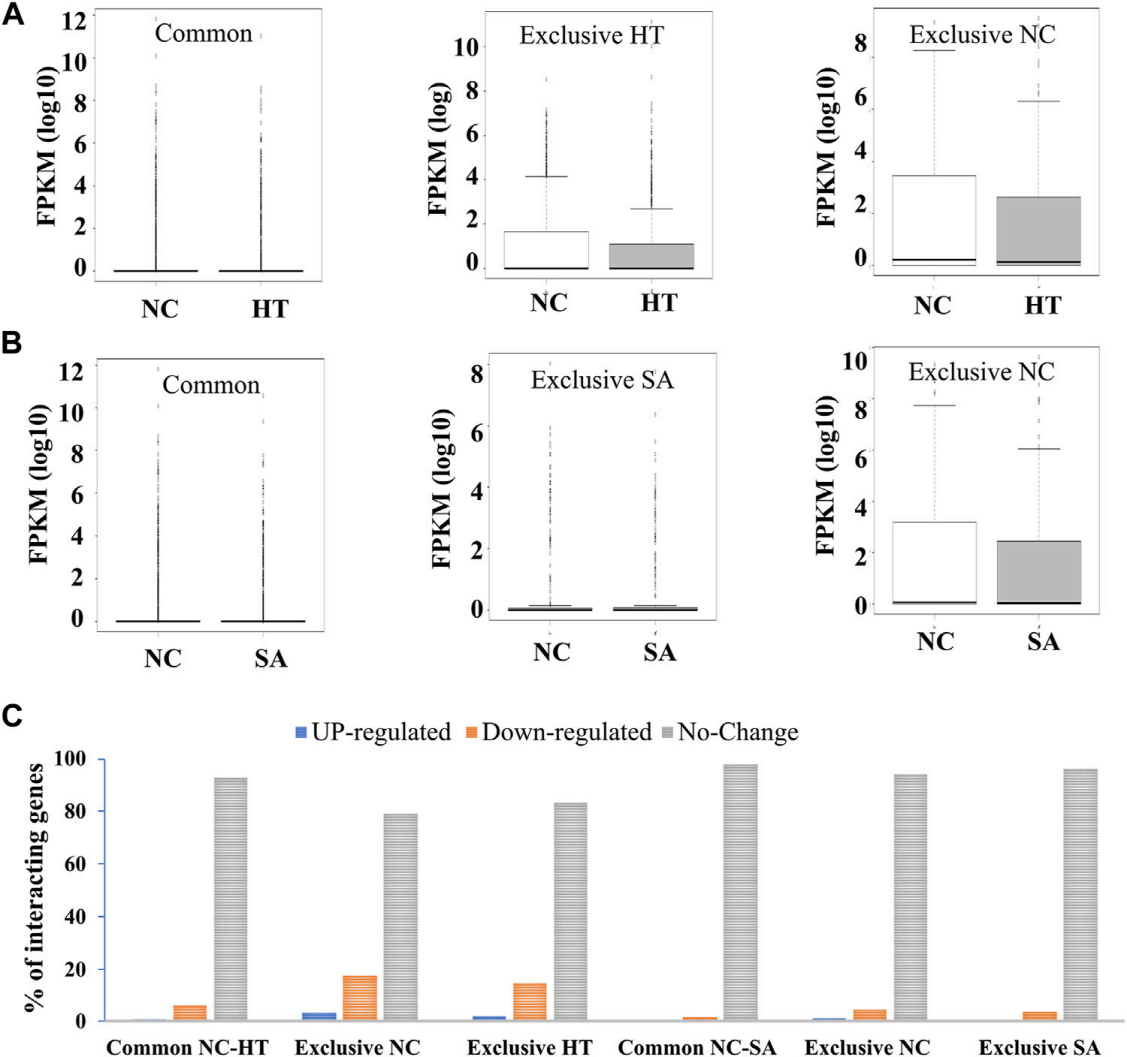
Expression profiling of common and uniquely interacting genes in control and stress conditions. Enrichment and depletion of interaction leads to common and unique interacting genes in HT as well as in SA libraries in comparison to the NC. These common and uniquely interacting genes in **(A)** NC vs. HT and **(B)** NC vs. SA do not show any significant change in the expression profile in control and treated samples (*p*-value >0.005) indicating that these interactions may not directly regulate the expression of interacting genes. **(C)** Bar chart showing the percentage distribution of up-regulated, down-regulated, and unchanged expression of interacting genes in the common and exclusive interacting regions.

Our analysis revealed common and exclusive interactions among all the interactions identified in three conditions, namely, NC, SA, and HT (Supplementary Figure S5). The intriguing question was whether these changes in interactions affect the expression of genes involved in those interactions. We thus compared the expression profiles of common and uniquely interacting genes involved in the interactions in untreated (NC) and treated (HT and SA) conditions. We found no significant difference in the expression profile of common and uniquely interacting genes in NC and SA and HT conditions (Figures 2A,B). Further, we analyzed the percentage distribution of up-regulated, down-regulated, and unchanged expression of interacting genes in the common and exclusive interacting genes (untreated vs. treated). This revealed that most of the genes involved in the interactions showed no change in the expression profile (Figure 2C). These results indicate that *Arabidopsis* responds to different treatment (HT and SA) by either enrichment or by depletion of chromatin contacts, but this change in contacts may not lead to the uniform change in global expression profile of interacting genes. Since we did not observe any significant difference in the expression pattern of common and uniquely interacting genes at high resolution (1 kb), we identify interacting regions at lower resolution (200 kb) to explore the difference in the expression profile of larger interacting blocks. We identified 399 interaction blocks shared between NC and SA, and 58 and 34 interaction blocks unique to NC and SA, respectively. Similarly, 440 interaction blocks were common in NC and HT, and 17 and 131 were unique to NC and HT, respectively. We next calculated the cumulative expression of all genes in common and unique blocks in treated and untreated conditions. However, even at a lower resolution of 200 kb, we did not observe any significant change in the expression of genes involved in the common interaction or unique interactions in different conditions (Supplementary Figure S6). Our results thus indicate that enrichment or depletion of chromatin contacts at lower or higher resolution does not directly influence the global gene expression. We hypothesize possibly that change in the contacts and thus gene expression may be limited to a few specific cells, and thus the analysis of global gene expression in entire seedlings does not reveal any change in the expression.

### Enrichment of Heterochromatin-Related Epigenetic Signature in Interacting Regions

**FIGURE 3:**
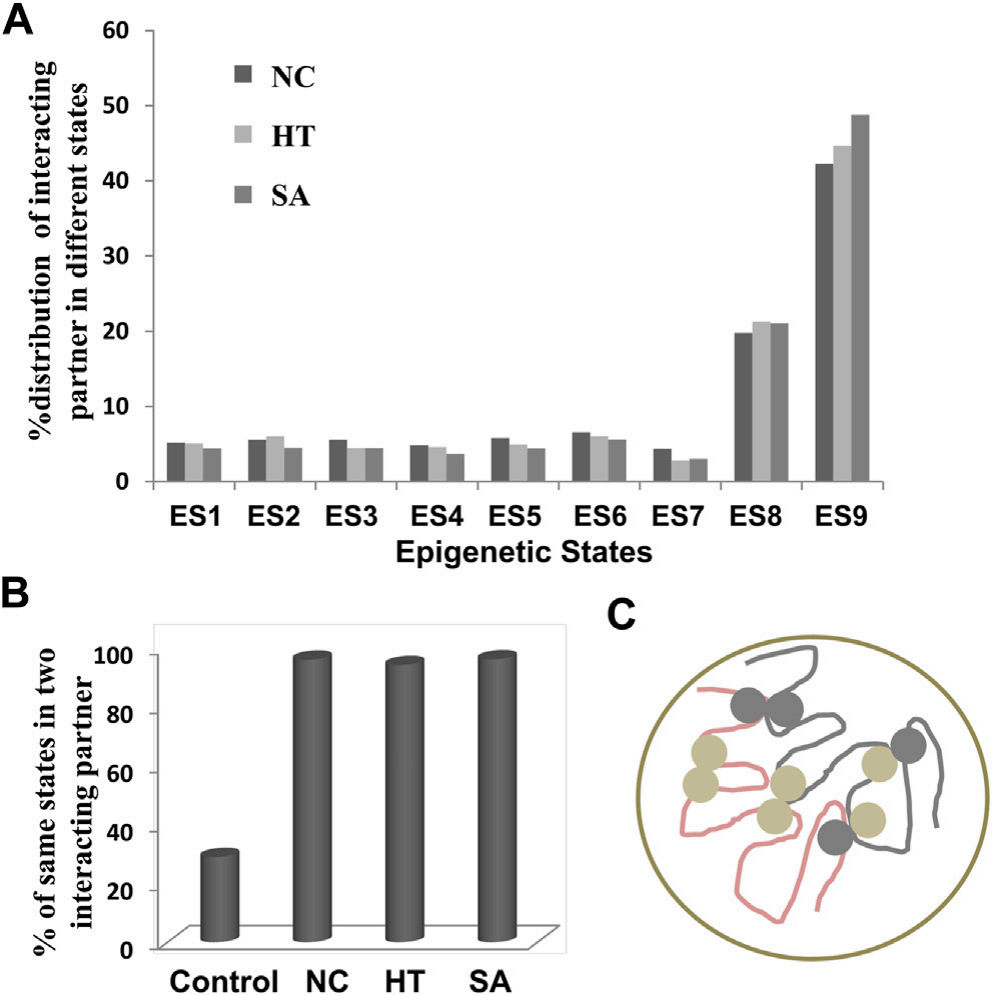
Distribution of interacting regions into various epigenetic states. **(A)** Mapping of interacting regions on different epigenetic states as defined previously (Sequeira-Mendes et al., 2014). Interacting regions were highly enriched in state 8 and 9 which are the marks for heterochromatic regions and the least represented in state 7 which is exclusively associated with the intragenic regions. **(B)** The interactions frequency of two interacting regions among the NC, HT, and SA libraries sharing the same ES is significantly high over the control sets of interacting regions (*p*-value <0.00001) indicating that interactions were firmly associated with the epigenetic states of interacting regions. **(C)** Cartoon representing the interactions among the same ES is more frequent than the different ES (light and dark grey circles represent the different ES).

Sequeira-Mendes and colleagues defined *A. thaliana* genome into nine ES based on various epigenetic marks (histone variants, histone marks, and CG methylation) (Sequeira-Mendes et al., 2014). Each ES has sets of enriched epigenetic marks and a few characteristic features. We mapped the coordinates of identified interacting regions to the ES to classify the identified interactions into various ES. The ES one to seven represent poorly in interactions, always less than 10% in all the conditions (Figure 3A). The distribution of interactions into various ES in all three conditions showed an almost similar pattern. We found significant enrichment of interacting regions in the ES8 (more than 20%) and 9 (more than 40%), these ES, marks for the heterochromatic region of the genome and enriched in epigenetic marks such as mCG, H3K9me2, and H3K27me1 (Sequeira-Mendes et al., 2014). The ES9 is almost twice enriched than ES8 in all three conditions in our analysis, although both represent the heterochromatic region but differ in the genomic position (Figure 3A). ES8 preferentially co-localizes with intergenic regions (AT-rich) while ES9 corresponds to heterochromatic pericentromeric regions (GC-rich). Both ES8 and 9 are highly enriched with the transposable element (TE); however, the enrichment of TE is comparatively higher in ES9 compared to ES8. The distribution of interacting elements in epigenetic ES8 and 9 indicates that identified interactions are mainly represented in the heterochromatin region. Since our chromatin interaction data over-represent TEs, we also analyzed a separate set of chromatin interactions excluding TEs. We identified that the distribution pattern of interacting regions without TEs into various ES, are more or less similar; however, the overall percentage of interacting regions that fall into ES8 and 9 was decreased but still higher than other ES (Supplementary Figure S7A). We identified that TEs involved in interactions showed more than 95% enrichment in ES8 and 9 (Supplementary Figure S7B). So, the above results indicate that not only the interacting TE but other interacting regions were also highly enriched in the ES8 and 9.

Next, we examined whether there are any preferential interactions among these ES. We identified the ES of both the interacting partners in NC, HT, and SA conditions and compared them with the randomly generated interactions used as a control. We found that interacting partners are always likely to be in the same ES which is always more than 90% instances in all the three conditions analyzed (Figures 3B,C). This enrichment of the same ES in the interacting partners was significantly higher (*p*-value < 0.00001) than the control set selected which showed merely 30% enrichment of the same ES (Figure 3B). The result indicates that chromatin interactions govern the folding principle of chromatin by bringing together similar epigenetic marks.

### Conserved Motifs in the Interacting Regions Impart in Suppression of Genes Expression

**FIGURE 4:**
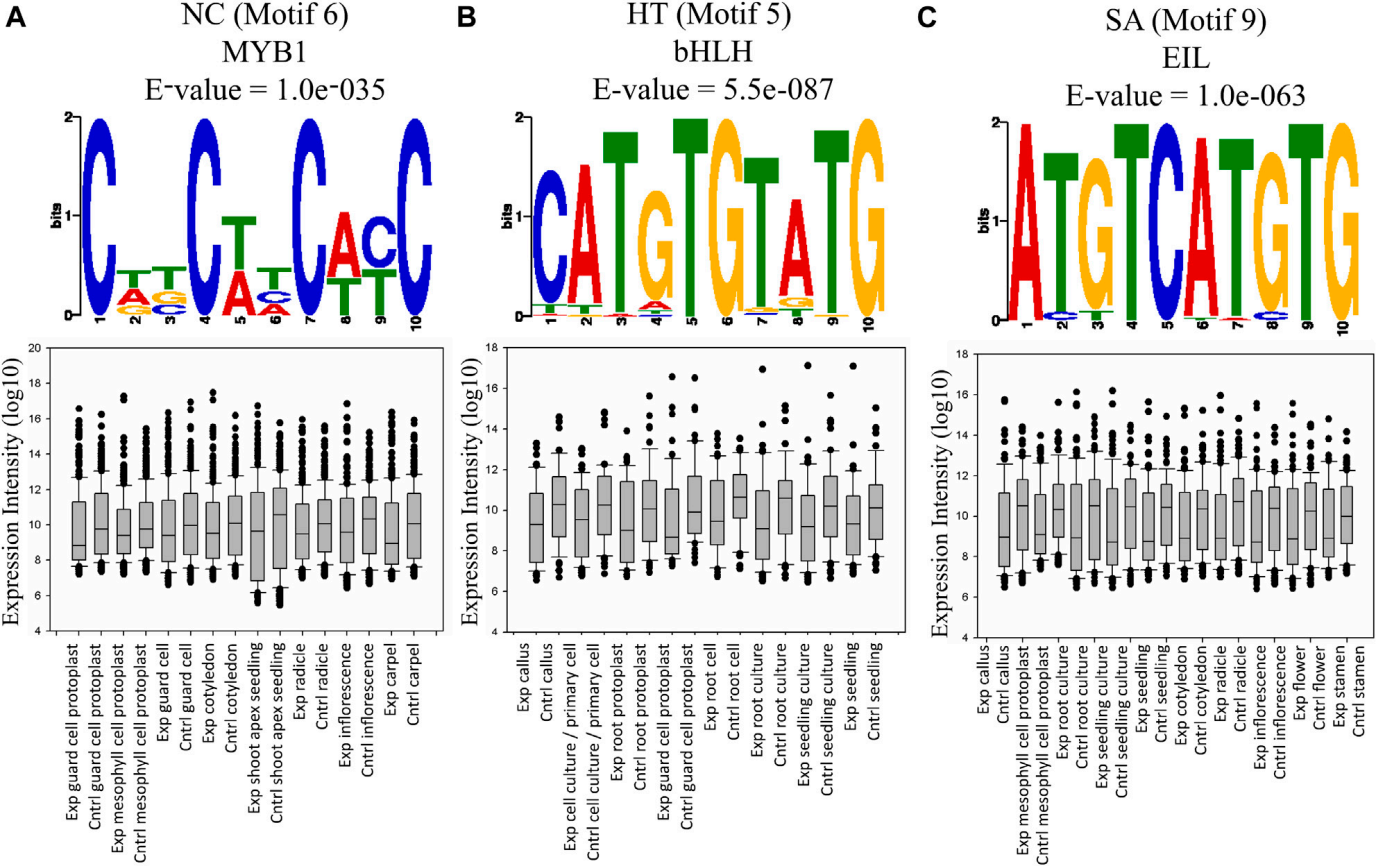
The expression profile of interacting protein-coding genes having the binding site for known TFs. In this figure, some of the conditions represented are showing a statistically significant difference over the control (*t*-test *p*-value < 0.05). Interacting genes containing motifs for different TFs showed significantly lower expression in different tissues or plant parts. **(A)** Motif 6 of NC. **(B)** Motif 5 of HT. **(C)** Motif 9 of SA.

We used MEME (Bailey et al., 2009) to identify the top 10 conserved motifs in those interacting sequences in which at least one partner was a protein-coding gene, in all three conditions (Supplementary Figure S8). Predicted motifs were further annotated for a *cis*-regulatory element with STAMP (Mahony and Benos, 2007) using the AGRIS database (The *Arabidopsis* Gene Regulatory Information Server, http://*Arabidopsis*.med.ohio-state.edu/). The motif containing AAGCTT was conserved in the top two positions among all the conditions (Supplementary Figure S9). The conservation of AAGCTT is expected since it is a HindIII site that is used for preparing the Hi-C libraries and thus it further validates the quality of our Hi-C library. Predicted motifs were annotated into 9 *cis*-regulatory elements in NC, HT, and SA libraries, respectively. Among these four regulatory elements, MYB1, ERE, SORLIP5, and LS7 were exclusively found in NC, while EIL1 and OBP-1_4_5 were exclusively present in SA, and LFY and AG_v3 were found in the HT library (Figure 4; Supplementary Figure S8). RAV1 element which provides the binding site for the TF Related-to-ABI3/VP1 (RAV) was identified in the NC and HT conditions, while the LFY and PRHA *cis*-regulatory elements have the binding site for Orphan and Homeobox TFs were found in HT and SA treated libraries. The very high conservation of some of the known TF binding sites in the interacting regions indicates the probable role of these elements in co-regulation of interacting genes. To explore this possibility, Motif RAV1-A and MYB1 for NC, Motif AtMYC2, PRHA, and LFY in HT and motif PRHA, and AtMYC2 and EIL1 in SA were selected further since these motifs are known to interact with known TFs (Supplementary Figure S9). We identified a set of genes interacting with each motif and also selected a random set of non-interacting genes as a control. The expression profiles of the interacting and non-interacting genes were retrieved from 69 cell and tissue conditions using the GENVESTIGATOR database. We identified for motif 4 and 6, there were 3 and 23 distinct expression profiles in NC, respectively, similarly for motif 5 and 8, 46 and 1 profiles in HT and motif 6, 7, and 9 in SA, 12, 58, and 37 profiles were the expressions of interacting genes was lower (*p*-value < 0.05) than the control dataset (Supplementary Figure S9). Thus, irrespective of the motif they are interacting, interacting genes showed either lower expression or no difference than non-interacting genes in specific cell and tissue conditions. Thus, results indicate that either these conserved motifs bind to TFs which are largely suppressor or TFs may activate genes specifically in restricted cell type or temporally or conditionally which cannot be identified by global expression profiling in set parameters.

### Interacting Regions Exclude Quantitative Trait Loci Associated with Phenotypic Diversity

**FIGURE 5:**
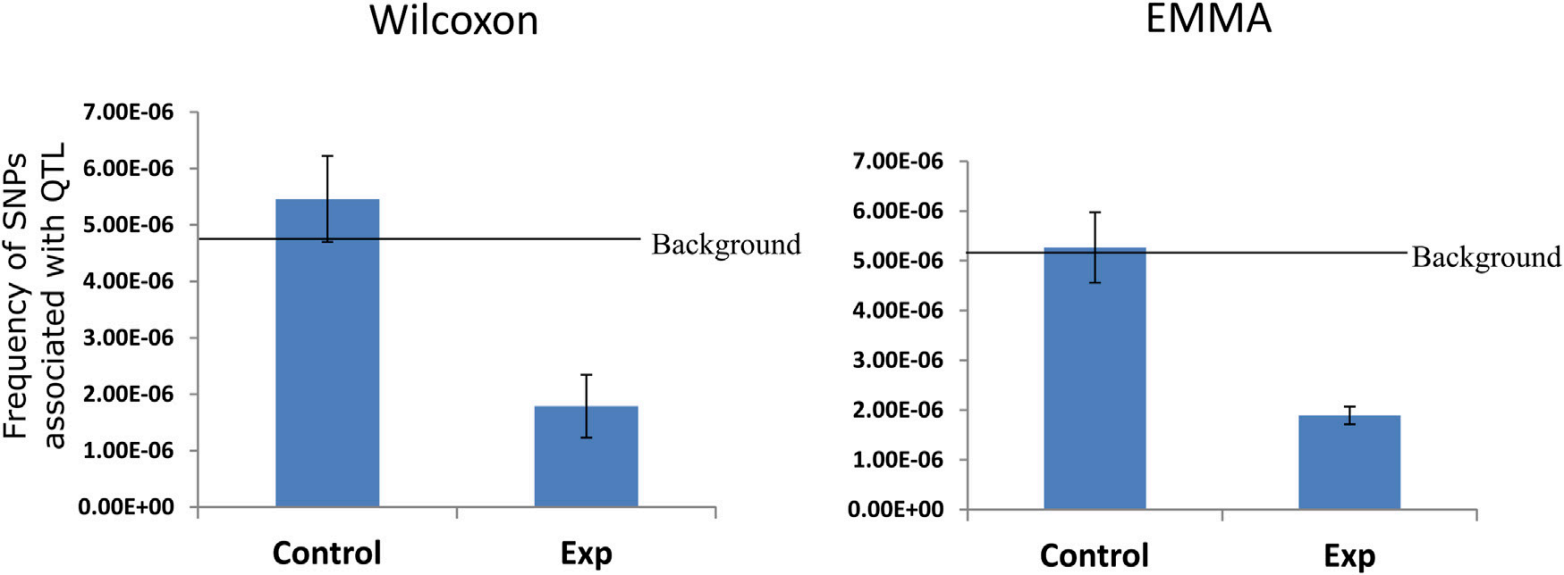
Distribution of SNP associated with QTL in interacting regions. Mapping of publicly available SNP (Atwell et al., 2010) associated with quantitative trait on interacting and non-interacting regions (control), showing more than two times depletion of GWAS hit in the chromatin interacting region over the non-interacting regions. The selected control region is not biased since genome-wide GWAS SNP frequency (background) is similar in the background and control region.

Further, we ask a question whether the chromatin interacting region has a functional role in controlling phenotypic diversity in *A. thaliana*. We made use of GWAS data (Atwell et al., 2010) which revealed using EMMA and Wilcoxon test 615 SNPs and 567 unique SNPs respectively associated with the quantitative trait loci for several phenotypic traits in natural accessions of *A. thaliana*. We calculated the frequency of association of SNPs in interacting genome; we also consider the same length of a non-interacting genome as a control and also the entire *A. thaliana* genome to calculate background frequency of associated SNP. The frequency of associated SNP in the non-interacting genome is marginally higher than the background frequency (Figure 5). However, interestingly the frequency of associated SNPs identified using EMMA and Wilcoxon test was almost two times lower in the interacting regions of *A. thaliana* genome (Figure 5). The result indicates that the SNPs associated with phenotypic diversity in the natural population of *A. thaliana* are preferentially excluded from the portion of the genome involved in chromatin interactions.

## Discussion

The establishment of Hi-C protocol (Lieberman-Aiden et al., 2009) leads to extensive work resulting in the identification of genome-wide chromatin interaction networks in human (Sandhu et al., 2012; Jin et al., 2013), *Drosophila* (Sexton et al., 2012), yeast (Duan et al., 2010), and plants (Moissiard et al., 2012; Grob et al., 2013; Grob et al., 2014; Feng et al., 2014; Wang et al., 2015; Liu et al., 2016). In the present work for the first time, we analyzed the effect of either hormonal treatment or abiotic stress on chromatin interaction networks.

As previously reported, we also observed that the nuclear architecture of *A. thaliana* is significantly different from others as its genome is not partitioned into the larger interactive topological domain (Moissiard et al., 2012; Feng et al., 2014). This spectacular feature could be explained due to the lack of CTCF in *Arabidopsis* genome (Heger et al., 2012). As previously reported, we have also identified that *A. thaliana* genome can be partitioned into two broad groups based on the correlation of interacting reads (Figure 1C) (Lieberman-Aiden et al., 2009; Sexton et al., 2012; Yadav et al., 2021). The correlated region has a higher frequency of interacting reads than the non-correlated region of the genome (Grob et al., 2014). Further, our analysis of correlation heat map showing the visual transition of chromatin compartment in HT condition (Figure 1C; Supplementary Figure S3). Similar observations have recently been reported for *Arabidopsis* and rice (Sun et al., 2020; Liang et al., 2021), suggesting that the compartment transition might be conserved among different species. We have uncovered several interactions in the heterochromatic and euchromatic regions that established physical communication with the different regions of the genome, which will provide a new mechanistic way of gene regulation in plants (Figure 1). Our interaction data suggest that the interactions along the chromosome are more frequent (∼69–74%) than those between the chromosomes (∼26–31%) (Supplementary Table S1) and interaction frequency is higher for interacting pairs of loci that lie close together in a linear chromosome (Figure 1E), which is the general feature of spatial chromatin organization (Lieberman-Aiden et al., 2009; Sanyal et al., 2012; Feng et al., 2014; Grob et al., 2014). We observed that heat stress induces the genome-wide long-range chromatin interactions (>20 Kb, ∼55 vs. ∼50% in HT vs. NC) but decreases short-range chromatin interactions (<20 kb, ∼45 vs. ∼50% in HT vs. NC; Supplementary File 3) which is contrasting to the previous observation in *Arabidopsis* (Sun et al., 2020). However, in rice it is reported that the short-distance interactions were decreased during heat stress (Liang et al., 2021). This variation could be technical or because of different development stages of plant material and heat stress condition used in both studies.

We found an increase in *trans* interactions (∼29.9 vs. ∼25.8% in HT vs. NC) after heat stress similar to a previous study in rice (Liang et al., 2021). Further, we also identified that the frequency of inter-chromosomal interaction is quite high in the pericentromeric regions (Figure 1D) which is a general feature of inter-chromosomal contact (Lieberman-Aiden et al., 2009; Feng et al., 2014; Grob et al., 2014; Wang et al., 2015; Nützmann et al., 2020). We assume the enrichment of inter-chromosomal interactions near the centromeric regions facilitated the heterochromatin-mediated chromatin fiber concretion at the centromere, providing structural constraints vital for the genome organization (Mizuguchi et al., 2014). Since we found substantial inter-chromosomal interactions in the pericentromeric regions which are generally enriched with crucial heterochromatin marks, it is not surprising that we observed enrichment of heterochromatic regions in the interactions. These results are in line with previously reported studies in *A. thaliana* (Moissiard et al., 2012; Feng et al., 2014; Grob et al., 2014; Yadav et al., 2021). Interactions between the heterochromatic regions and genes possibly assist the cell or organ-specific clustering of interacting genes hence regulating the expression in specific tissue type (Fransz et al., 2002; Nützmann et al., 2020; Yadav et al., 2021). Recently, Nutzmann and colleagues demonstrated that dynamic clustering of biosynthetic gene cluster with chromatic arm (euchromatic region) and pericentromeric (heterochromatic region) in root and leaf, respectively, define the transcriptional active and repressed states (Nützmann et al., 2020). Furthermore, intra- and inter-chromosomal contacts between the pericentromeric regions of the different chromosomes are high which is enriched with the TE, thus these interactions may be playing an important role in keeping transposons silent and thus maintaining the integrity of the genome (Grob et al., 2014). We also identified interactions among the telomere of different chromosomes and telomere with the centromere as reported earlier (Moissiard et al., 2012; Feng et al., 2014; Grob et al., 2014) and which is in concordance with previous DNA FISH assay (Fransz et al., 2002; Schubert et al., 2012).

Uniquely, we were interested in addressing whether a stress condition alters the number of significantly identified interactions and changes in the interactions influence the expression of genes associated with it. Our results revealed that a stress condition either biotic (as mimicked by SA) or abiotic does result in the change in the chromatin interactions (Supplementary Figure S5). The stress condition leads to establishment of 896 and 2789 new interactions and loss of 1222 and 1104 interactions in SA and HT treatment, respectively, as compared to native conditions (Supplementary Figure S5). The chromosomal reorganization in response to heat stress is consistent with the previous studies in *Arabidopsis* and rice (Sun et al., 2020; Liang et al., 2021). Our data show that the chromosomal contact increased in response to heat stress compared with the control (Supplementary Table S1). Similar observations have been reported for *Arabidopsis* seedling demonstrating that in general the chromosomal contact is enhanced between different regions along the chromosomes (Sun et al., 2020).

Our results at 1 Kb or 200 Kbs revealed that whether the interactions are enriched or depleted due to stress conditions at any loci does not affect the global gene expression associated with interactions (Figure 2; Supplementary Figure S6). This result brings us to an important question that if not for the accommodating change in the expression of genes associated with interactions, then why was there so much change in the interactions profile after stress treatment? The possibility could be the chromosomal organization is prone to random variation which is unlikely caused by essentially biological processes hence cannot directly correlate with the transcriptional state of the cell (Nagano et al., 2013; Grob et al., 2014). It should also be noted that we are likely to underestimate the impact of interactions on gene expression because captured interactions are not happening in all the cells and tissues and might be representing a particular type of cell and tissue. Since we analyzed the expression in the seedlings this does not directly reflect those finer changes. However, it does not remain a limitation when working with the animal cell lines or cell/tissue specific analysis where the expression is directly correlated with the interactions (Chepelev et al., 2012; Dixon et al., 2012; Jin et al., 2013; Yadav et al., 2021). Thus, further detailed studies are needed to address such questions as to why chromatin interactions change after encountering stress conditions.

Mapping of interactions on previously identified ES (Sequeira-Mendes et al., 2014) in *A. thaliana* revealed that the interactions are highly enriched in repressive heterochromatin marks (H3K9me2, H3K27me1) is also in concordance with the recent report (Liu et al., 2016). It is also noteworthy that as reported previously (Feng et al., 2014) that H3K9me2 and H3K27me1 marks are highly enriched in interacting regions, our analysis also showed enrichment of ES8 and ES9 (Figure 3A) in the interacting *A. thaliana* genome which is distinctly enriched with H3K9me2 and H3K27me1 (Sequeira-Mendes et al., 2014). This further confirms that genes involved in the interactions are enriched with heterochromatin. Further, the ES of the interacting partners is likely to be the same (Figure 3B) which further confirms the folding principle of chromatin.

The possibility of interactions involving chromatin loops and regulating gene expression by either activation or suppression leads us to identify conserved *cis*-regulatory elements in the interacting region. The interacting regions showed significant conservation of binding sites of some of the characterized TFs (Supplementary Figure S8). These TFs play a major role in various plant growth and developmental processes, like bHLH in the regulation of a multiplicity of transcriptional programs (Toledo-Ortiz et al., 2003), RAV-1 was known to be a negative regulator of growth and its level repressed by hormones involved in abiotic stress (Fu et al., 2014). MYB TFs family participates in the regulation of various biological processes like responses to abiotic and biotic stress, metabolism, development, and differentiation (Ambawat et al., 2013). EIL family is involved in ethylene signaling in plants (Solano et al., 1998). This intriguing possibility is that these motifs might bind to suppressor or activator conditionally and regulate their expression by looping. It is also noteworthy to mention that the identified interacting genes are enriched from the heterochromatic and pericentromeric region and the control sets of genes are selected randomly and from the gene pools so this lower expression in different tissue could be technical instead of biological.

The previous studies (Moissiard et al., 2012; Feng et al., 2014; Grob et al., 2014; Liu et al., 2016) pointed out a folding principle that partitioned *A. thaliana* genome into highly interacting silenced heterochromatin region and less interacting active euchromatic region. We asked whether the interacting heterochromatin region is at all responsible for functional phenotypic diversity in *A. thaliana* occurs naturally in ecotypes. Interestingly, we observed that always QTLs associated with phenotypic diversity in a natural *A. thaliana* population are selectively excluded from the portion of the genome involved in the chromatin interactions (Figure 5). This is in a way not surprising considering most of the euchromatin and actively transcribing portion of the genome is excluded from the interaction. This raised very important questions about the evolution, functionality, and importance of these chromatin interacting regions that need to be addressed in the future.

## Data Availability

Sequence data generated for this study have been submitted to the NCBI under the BioProject ID: PRJNA317803 (Hi-C) and PRJNA317804 (RNA-seq).

## Supplementary Material

Supplementary file 1: Supplementary Table S1. Identified significant interactions at 1Kb resolution and its classification based on the location of interacting regions on chromosomes.

Supplementary file 2: Supplementary Figures S1-S9. S1. Genome-wide normalized interaction matrix of A. thaliana for HT and SA library. **S2**. Chromosome-wise contact matrices. **S3**. Genome-wide correlation interaction matrix. **S4**. Visualization of statistically significant interactions. **S5**. Venn diagram showing the common and unique interactions among different libraries. **S6**. Effect of interactions on gene expression at lower resolution (200kb). **S7**. Distribution of interacting regions into various ES. **S8**. Motifs identification and annotation. **S9**. Comparison of expression profile of interacting protein coding genes having the binding site for know TFs as identified through AGRIS annotation of motifs.

Supplementary file 3. Identification of significant interactions at 1 Kb resolution using HOMER.

Supplementary file 4. Hi-C libraries preparation and sequencing.

Supplementary file 5. Expression data of interacting genes in NC, HT and SA condition RNA-seq.

## Abbreviations

ES, epigenetic state; GWAS, genome-wide association study; HT, high temperature; NC, native condition; QTL, quantitative trait loci; SA, salicylic acid; TFs, transcription factors.
