# Supplementary material for: Stress Conditions Modulate the Chromatin Interactions Network in Arabidopsis
Source: Front Genet. 2022 Jan 5;12:799805. doi: 10.3389/fgene.2021.799805 (PMC8766718; doi:10.3389/fgene.2021.799805)
Supplement: Supplementary file 1 [file DataSheet2.PDF]

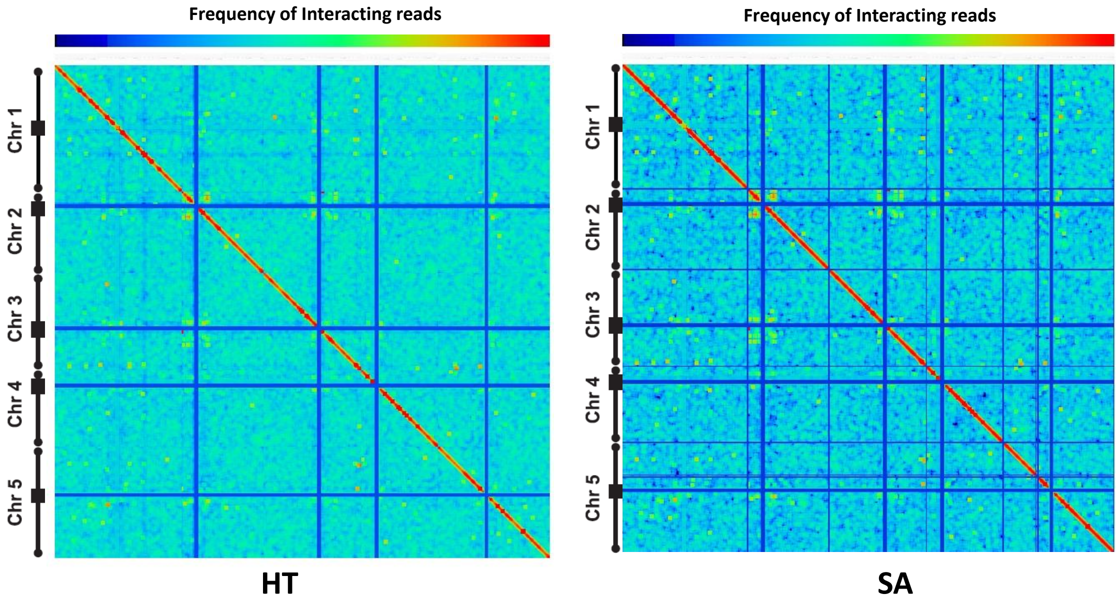

Fig. S1. Genome-wide normalized interaction matrix of *A. thaliana* for HT and SA library. Heat map showing an intense red diagonal line representing the enrichment of interacting reads at 200 kb resolution. Blue line representing reads that are less enriched in the centromeric and telomeric region of the genome. Colour bar ranging from blue to red represents the lower to higher enrichment of interacting reads. The black line representing the five different chromosomes in which rectangular box represent the pericentromeric and circle represent the telomeric region of the genome.

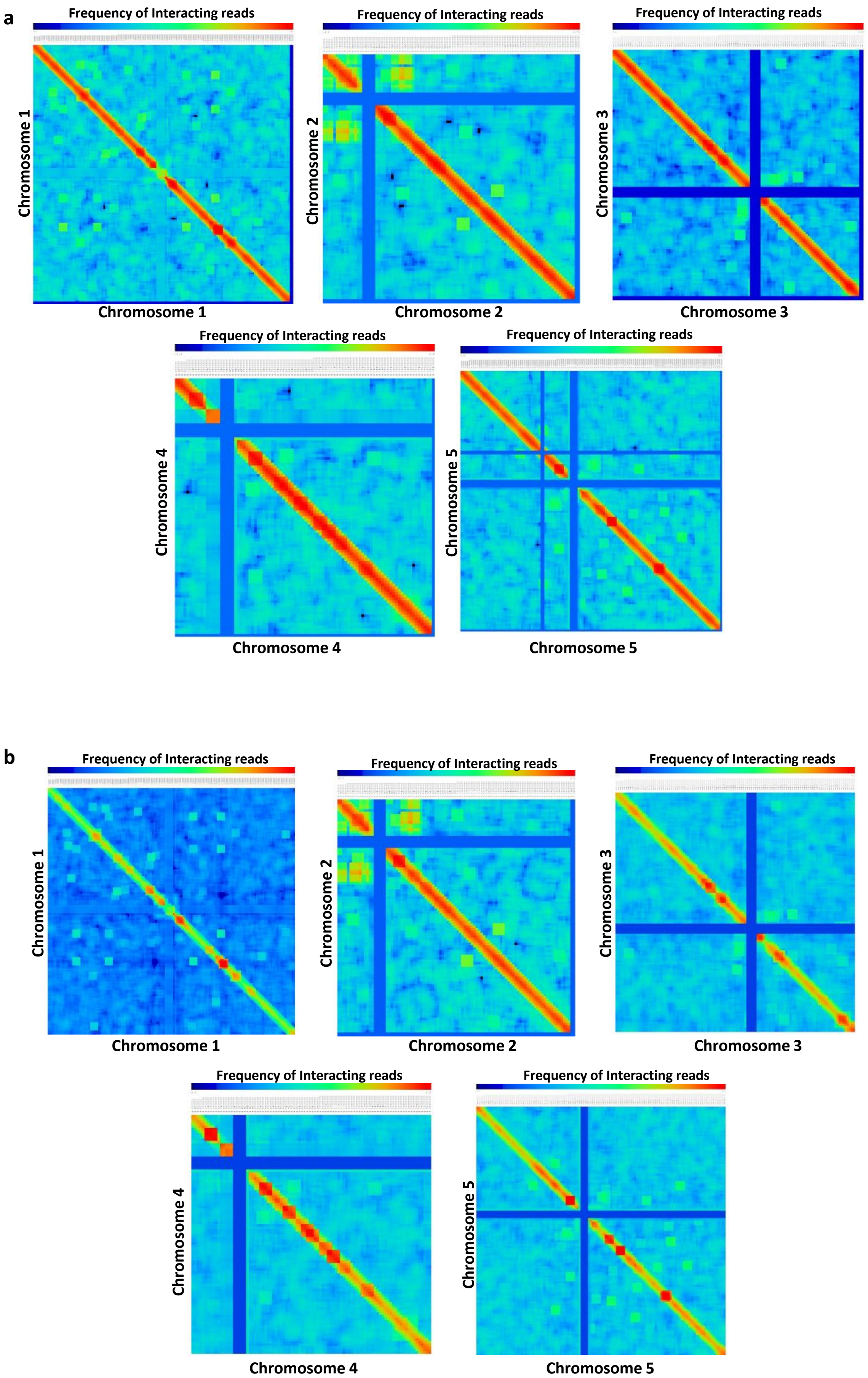
a
Frequency of Interacting reads
Chromosome 1
Chromosome 1
Frequency of Interacting reads
Chromosome 2
Chromosome 2
Frequency of Interacting reads
Chromosome 3
Chromosome 3
Frequency of Interacting reads
Chromosome 4
Chromosome 4
Frequency of Interacting reads
Chromosome 5
Chromosome 5
b
Frequency of Interacting reads
Chromosome 1
Chromosome 1
Frequency of Interacting reads
Chromosome 2
Chromosome 2
Frequency of Interacting reads
Chromosome 3
Chromosome 3
Frequency of Interacting reads
Chromosome 4
Chromosome 4
Frequency of Interacting reads
Chromosome 5
Chromosome 5

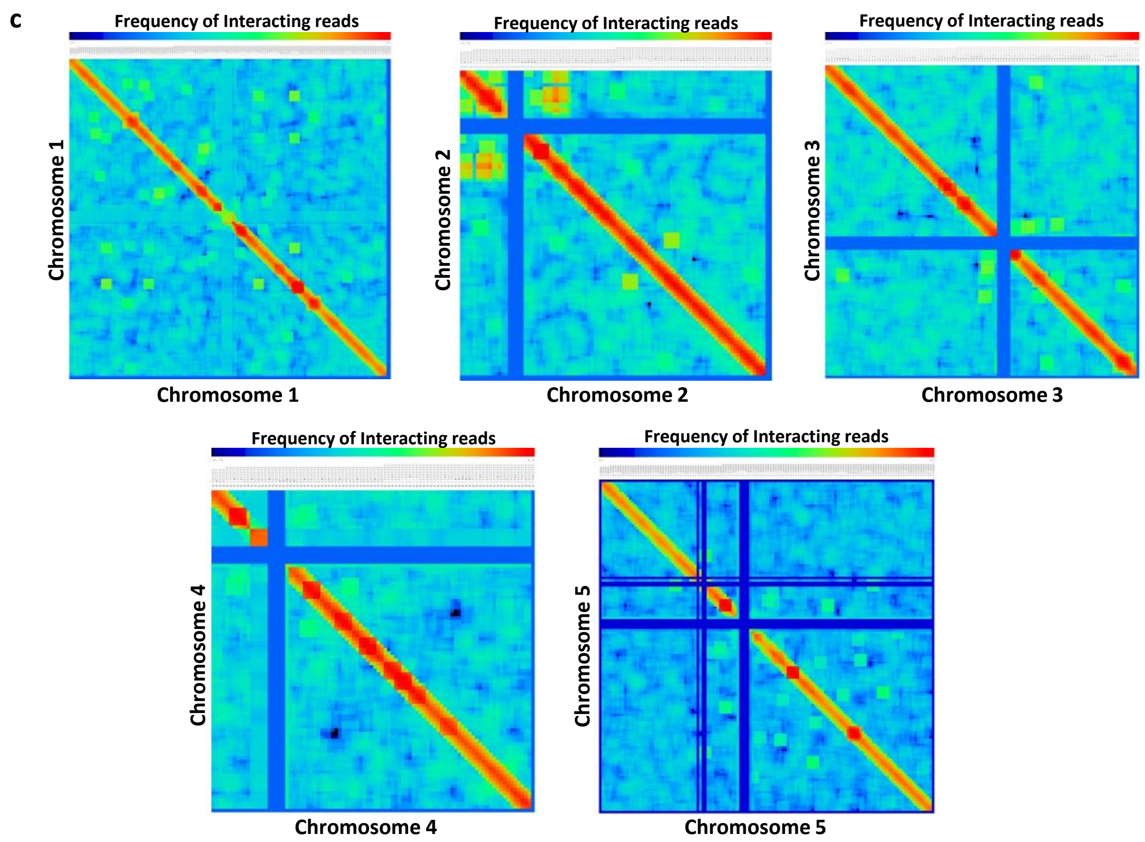

Fig. S2. Chromosome-wise contact matrix. The normalized interaction matrix for chromosome 1 to 5 shows many spots representing the enrichment of interacting reads in the **(a)** NC, **(b)** HT and **(c)** SA libraries. Enrichment spots indicate the presence of chromatin interactions over a long distance. Colour bar ranging from blue to red represents the lower to higher enrichment of interacting reads.

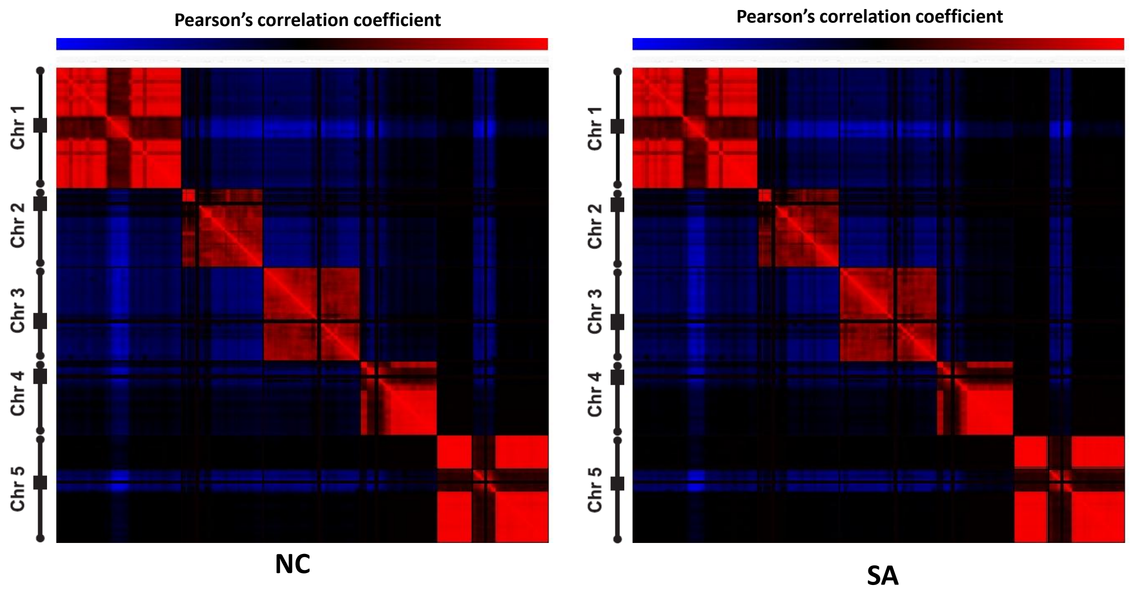

Fig. S3. Genome-wide correlation interaction matrix. Correlation interaction matrix at 200kb resolution representing the correlation coefficient among the interacting region in NC and SA libraries. It suggests the genome have two distinct regions positively correlated regions showing enriched reads and negatively correlated regions with depleted reads. Red colour represents the positive correlation and blue colour represents the negative correlation between the two regions.

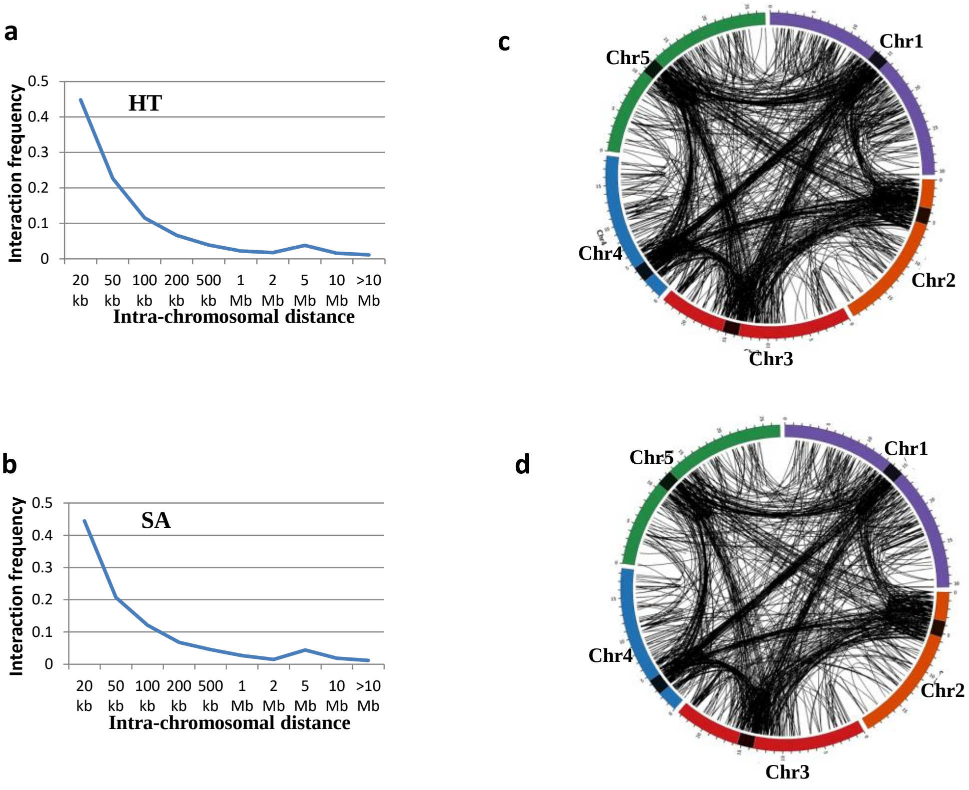

Fig. S4. Visualization of statistically significant interactions. (a and b) Graph showing the relationship of interactions frequency with the linear physical distance along the chromosome. The intra-chromosomal interaction frequency decreases with increasing linear distance on chromosomes (HT and SA). **(c and d)** Circos representing the genome-wide identified the significant interaction of HT and SA library respectively. Interactions were represented on the genome through the black line connecting the two points (interacting regions). Spans that link the regions within the chromosome represent the *cis* interactions while spans that link the regions between the chromosomes represent the *trans* interactions. The outermost coloured circle is the graphical representation of *A. thaliana* chromosomes and black rectangular box on it represent the centromeric region of each chromosome. Chromosome numbers are indicated after the chromosome (Chr) abbreviation.

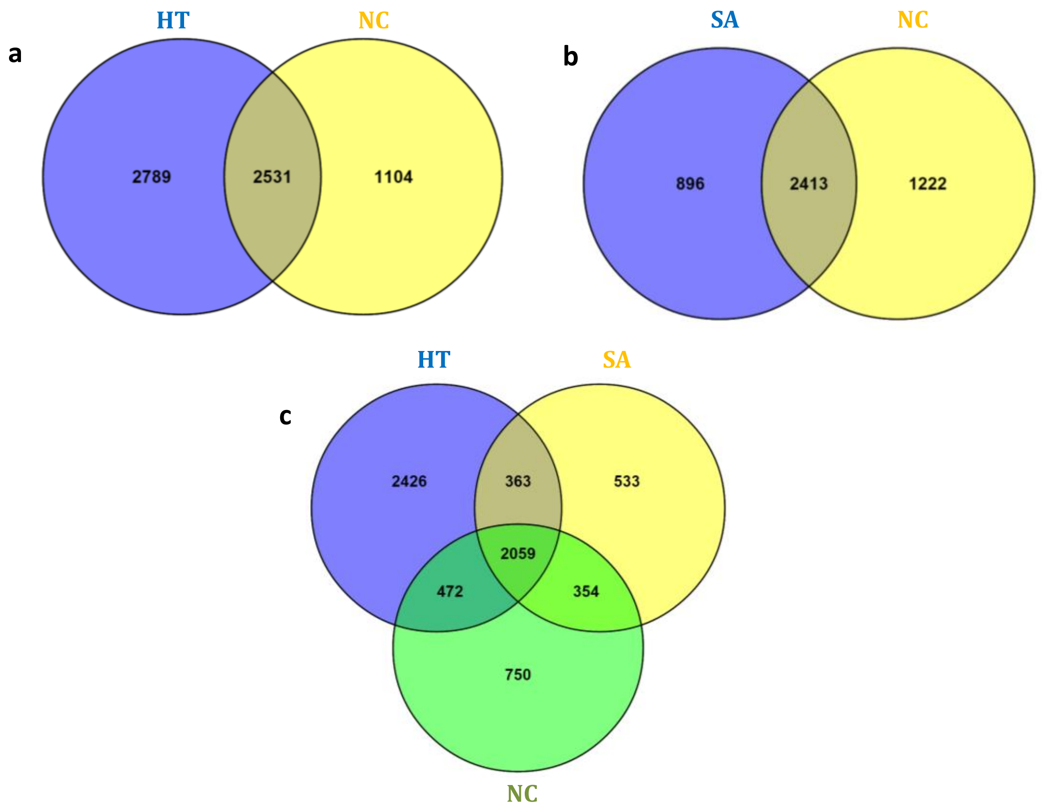

Fig. S5. Venn diagram showing the common and unique interactions among different libraries. Common and uniquely interactions were identified based on overlapping interacting regions. **(a)** 2531 interactions were common in NC and HT conditions while 1104 and 2789 interactions were unique to NC and HT condition respectively. **(b)** 2413 interactions were common in NC and SA conditions while 1222 and 896 interactions were unique to NC and SA conditions respectively. **(c)** 2059 interactions were common in all the three (NC, HT and SA) conditions while 750, 2426 and 533 interactions were exclusively unique to NC, HT and SA conditions respectively.

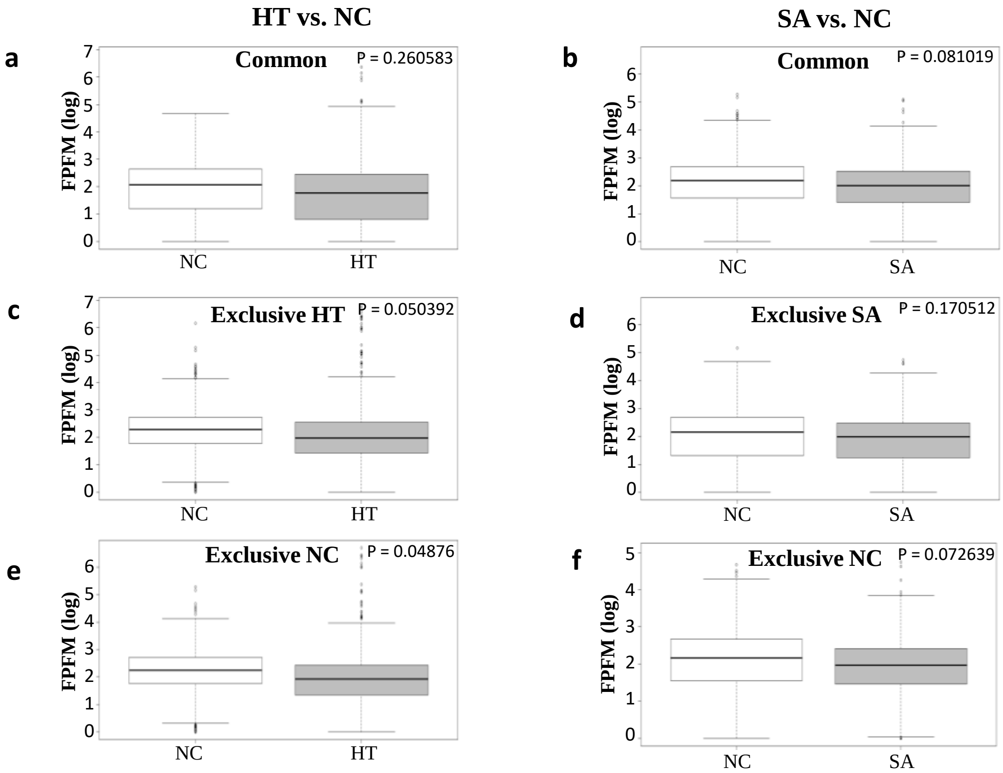

Fig. S6. **Effect of interactions on gene expression at a lower resolution (200kb).** The common and uniquely interacting regions at 200kb resolution in NC, HT and SA conditions were identified and their expression values were calculated and compared. These interacting regions in HT vs NC and SA vs NC do not show any significant difference in the expression profile in differentially interacting regions in NC and treated conditions. Common interacting regions between **(a)** HT and NC, **(b)** SA and NC. **(c)** Exclusively present in HT. **(d)** Exclusively present in SA. **(e)** Exclusively present in NC with respect to HT. **(f)** Exclusively present in NC with respect to SA.

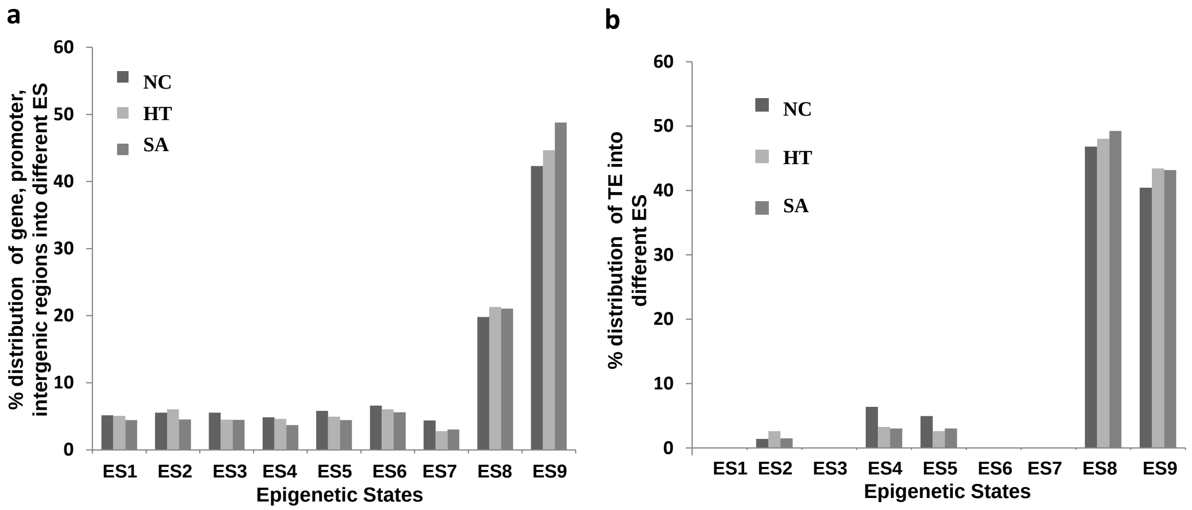

Fig. S7. Distribution of interacting region into various ES. (a) Distribution of interacting regions into various epigenetic states excluding transposable elements. Interacting regions were highly enriched in the state 8 and 9 which are the marks for heterochromatic regions and least represented in state 7 which is exclusively associated with the intragenic regions. **(b)** Distribution of interacting transposable elements into various epigenetic states. Interacting transposable elements were highly enriched in the state 8 and 9 which are the marks for heterochromatic regions and a very little portion is represented in state 2, 4 and 5.

a

S. No	Motifs in NC	E-value	Sites	Width	AGRIS Annotation	TFs binding Sites
1.	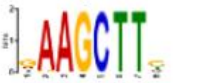	2.1e-267	1127	8	CCA1	MYB-related
2.	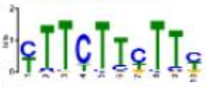	2.1e-233	1195	10	CCA1_v3	None
3.	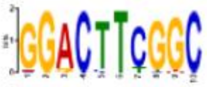	1.6e-038	64	10	T-box	None
4.	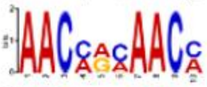	2.1e-021	197	10	RAV1-A	RAV
5.	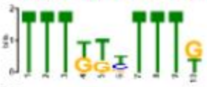	1.1e-096	586	10	PII	None
6.	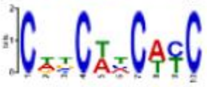	1.0e-035	624	10	MYB1	MYB
7.	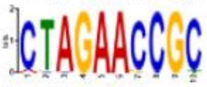	6.8e-028	54	10	ERE	None
8.	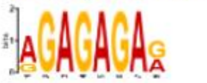	1.3e-027	357	8	SORLIP5	None
9.	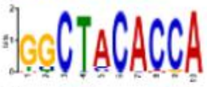	4.0e-022	79	10	LS7	None
10.	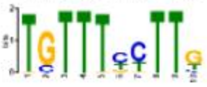	9.9e-013	327	10	CCA1_v3	None

b

S. No	Motifs in SA	E-value	Sites	Width	AGRIS Annotation	TFs binding Sites
1.	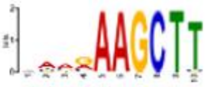	1.3e-312	1003	10	CCA1	MYB-related
2.	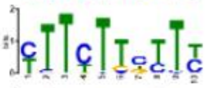	3.3e-161	1017	10	CCA1_v3	None
3.	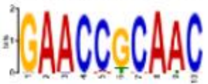	5.4e-157	142	10	octamer	None
4.	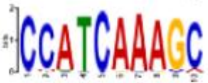	3.2e-133	171	10	SORLIP4	None
5.	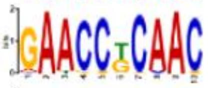	4.5e-116	159	10	octamer	None
6.	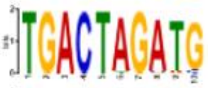	1.3e-111	135	10	PRHA	Homeobox
7.	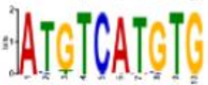	2.2e-104	135	10	AtMYC2	bHLH
8.	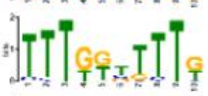	4.2e-057	908	10	PII	None
9.	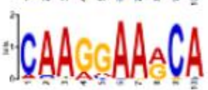	1.0e-063	265	10	EIL1	EIL
10.	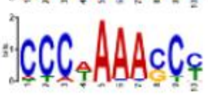	3.5e-036	320	10	OBP-1_4_5	None

c

S. No	Motifs in HT	E-value	Sites	Width	AGRIS Annotation	TFs binding Sites
1.	+ - 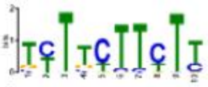	3.2e-322	1483	10	CCA1_v3	None
2.	+ - 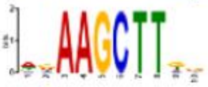	5.5e-363	1302	10	T-box	None
3.	+ - 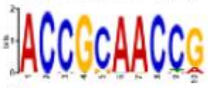	2.3e-139	146	10	RAV1-A	RAV
4.	+ - 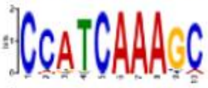	1.9e-117	181	10	SORLIP4	None
5.	+ - 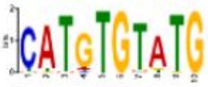	5.3e-087	244	10	AtMYC2	bHLH
6.	+ - 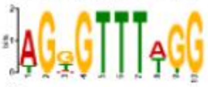	2.1e-074	215	10	LFY	Orphan
7.	+ - 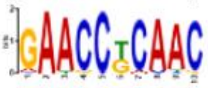	1.5e-109	201	10	octamer	None
8.	+ - 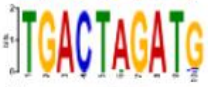	5.3e-103	132	10	PRHA	Homeobox
9.	+ - 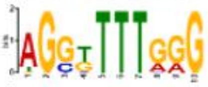	5.4e-055	250	10	LFY	Orphan
10.	+ - 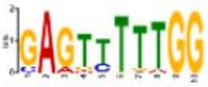	4.7e-074	255	10	AG_v3	None

Fig. S8. Motif identification and annotation. Top 10 enriched motifs conserved in the interacting region (1kb) involving protein coding genes using MEME search for **(a)** NC, **(b)** SA and **(c)** HT libraries. These significantly enriched motifs were annotated using AGRIS database to identify the conserved *cis*-regulatory element and also scan for the binding site for known TFs.

a

HT, Motif 5 (AtMYC2)

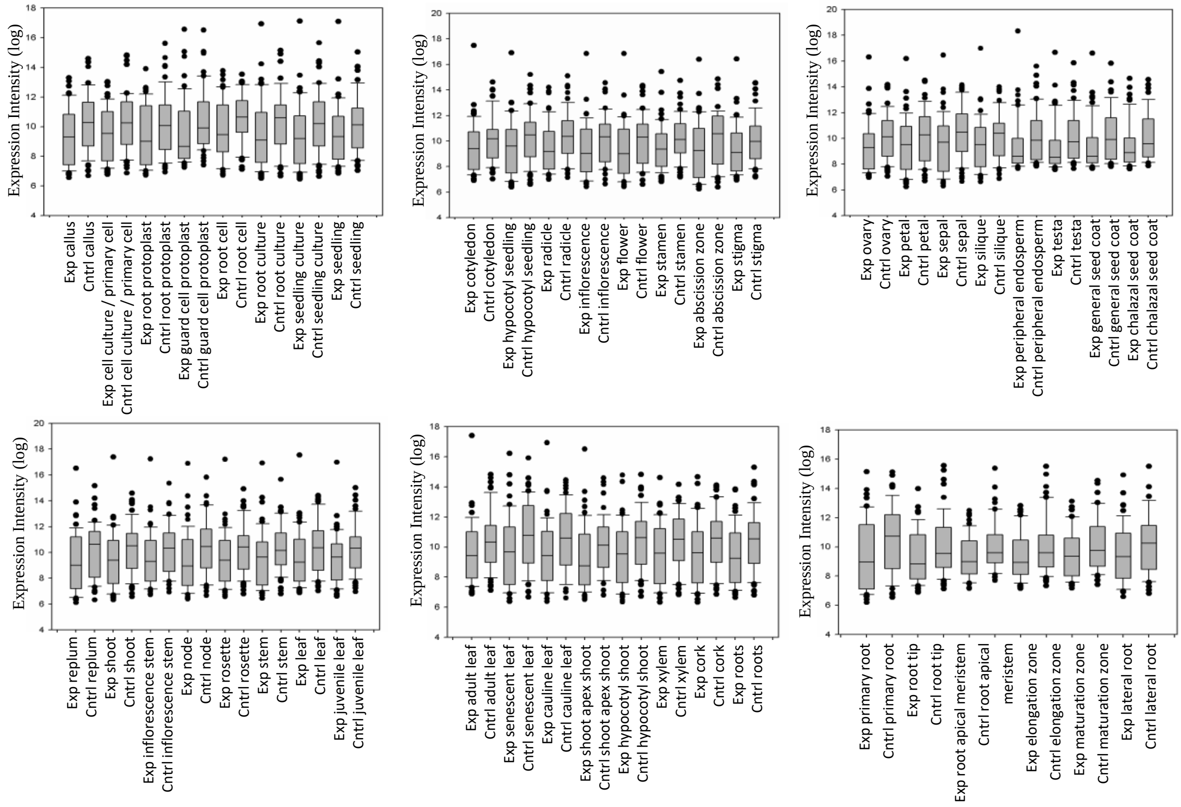

b

HT, Motif 8 (PRHA)

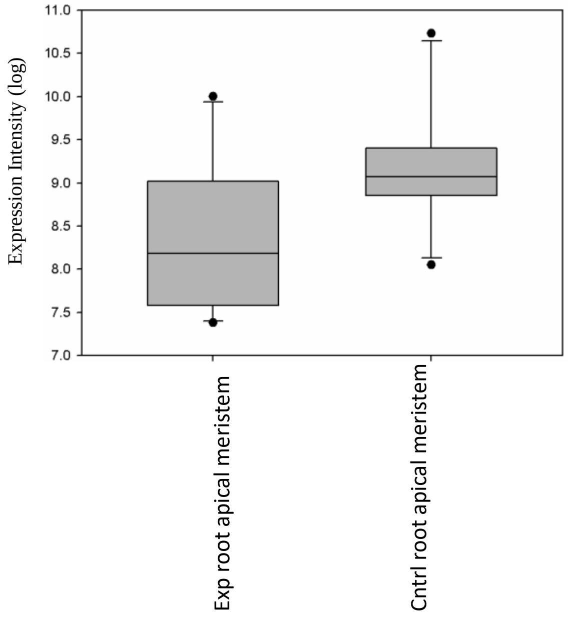

c

SA, Motif 6 (PRHA)

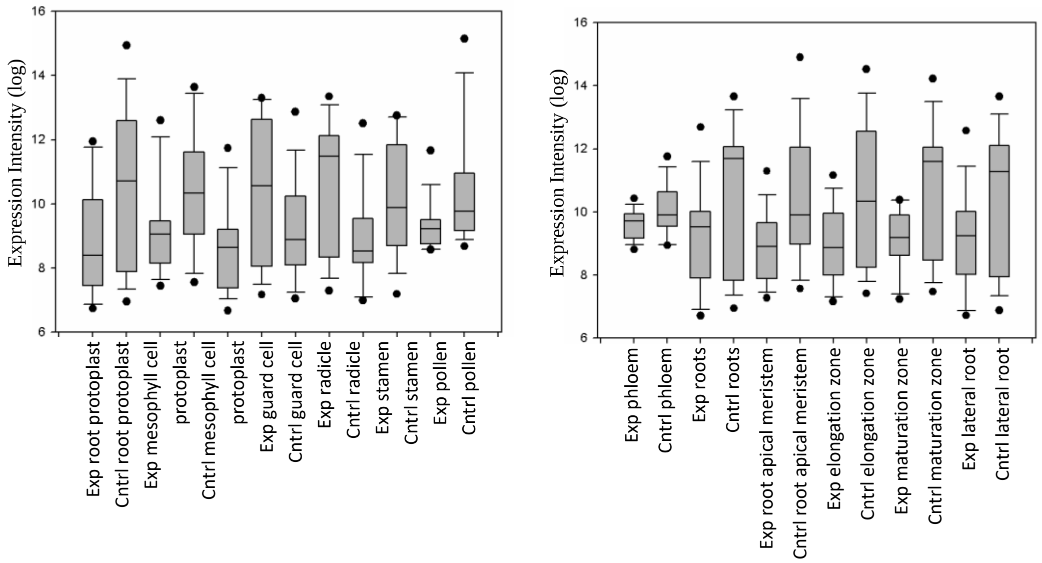

d

SA, Motif 7 (AtMYC2)

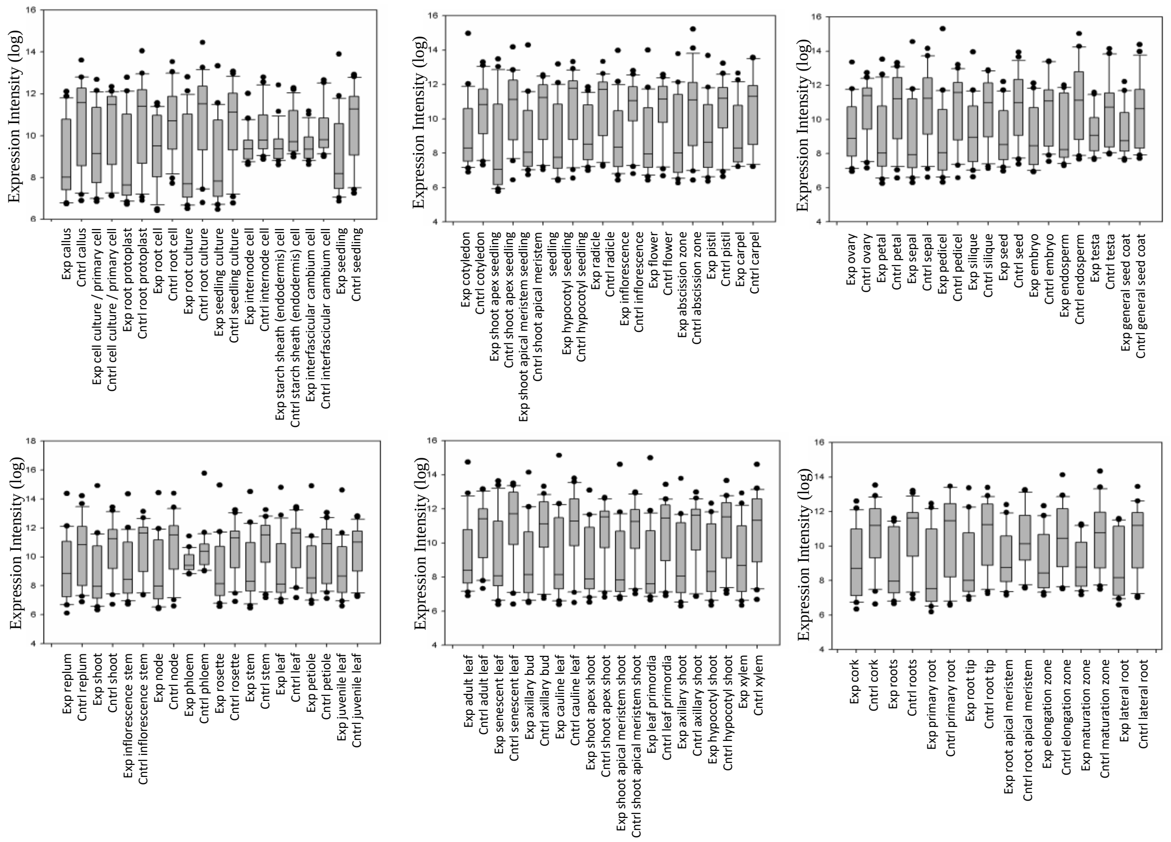

e

SA, Motif 9 (EIL1)

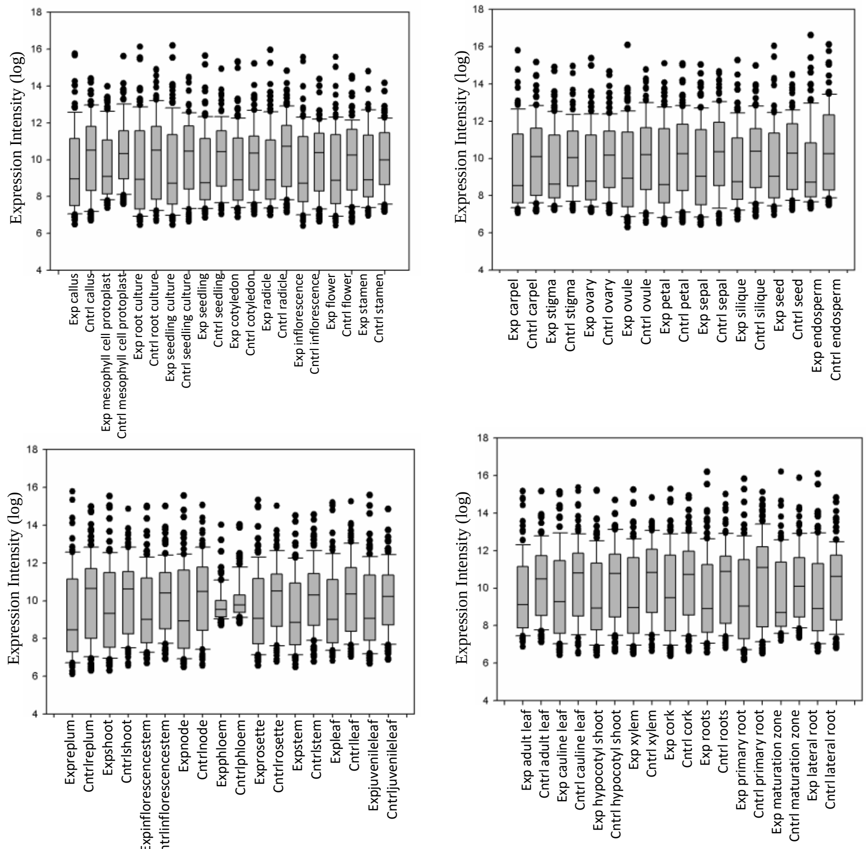

f

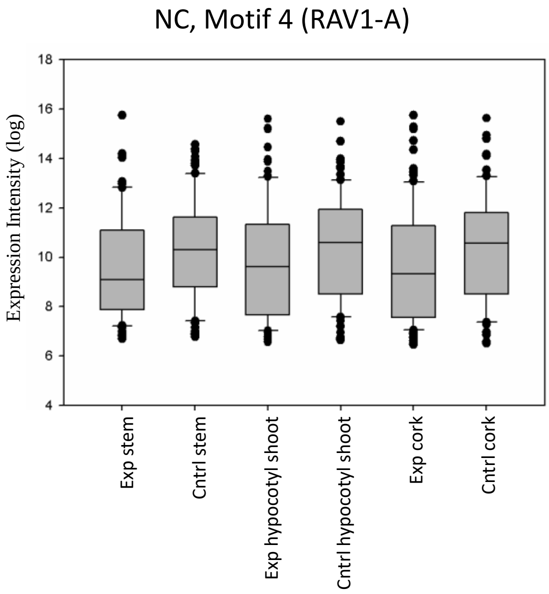
NC, Motif 4 (RAV1-A)
Expression Intensity (log)
18
16
14
12
10
8
6
4
Exp stem
Cntrl stem
Exp hypocotyl shoot
Cntrl hypocotyl shoot
Exp cork
Cntrl cork

g

NC, Motif 6 (MYB1)

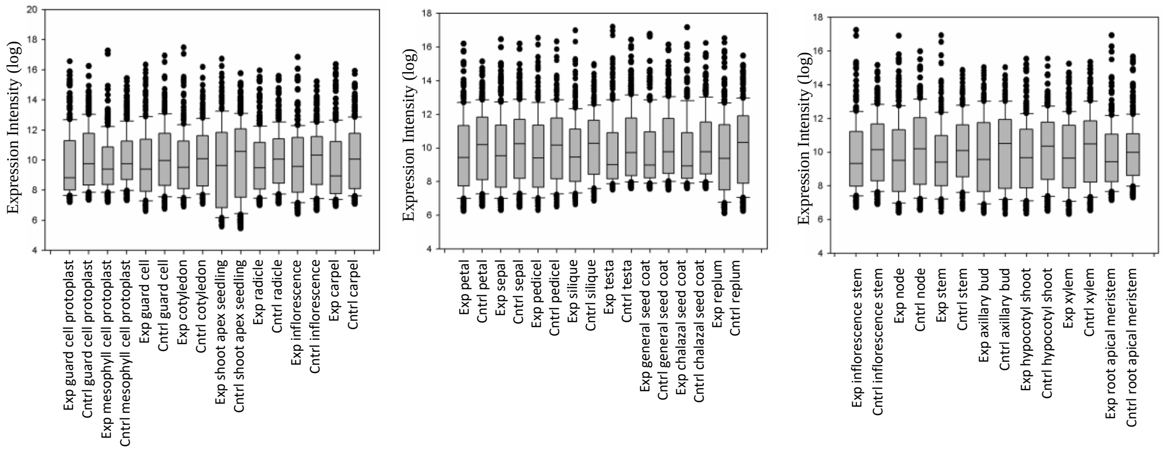

Fig. S9. Comparison of the expression profile of interacting protein coding genes having the binding site for know TFs as identified through AGRIS annotation of motifs. In figure expression of only those conditions represented which are showing a statistically significant difference over the control (t-test p-value < 0.05). Interacting genes containing motifs for different TFs show significantly lower expression in different tissue or plant parts in comparison to control. **(a)** Motif 5 of HT **(b)** Motif 8 of HT **(c)** Motif 6 of SA **(d)** Motif 7 of SA. **(e)** Motif 9 of SA. **(f)** Motif 4 of NC. **(g)** Motif 6 of NC.
