# Supplementary material for: Stress Conditions Modulate the Chromatin Interactions Network in Arabidopsis
Source: Front Genet. 2022 Jan 5;12:799805. doi: 10.3389/fgene.2021.799805 (PMC8766718; doi:10.3389/fgene.2021.799805)
Supplement: Supplementary file 3 [file DataSheet3.PDF]

Hi-C libraries preparation and sequencing.

First of all, nuclei were washed twice with 500 µl ice-cold 1x NEBuffer 2 (NEB). The pellet was then resuspended in 1.2x NEBuffer 2 in a total volume of 500 µl per tube. To inactivate the endogenous nuclease 20% SDS (final conc. 0.3%) was added per tube and the mixture was resuspended and incubated at 65°C for 20 minutes followed by 40 min at 37 °C while shaking at 200 rpm. SDS hampers in the restriction digestion hence quench using 10% Triton X-100 (cat no. 93443, Sigma) to a final conc. 2% was added and mixed carefully. Nuclei were subsequently digested overnight at 37°C by adding 400 Units HindIII (NEB) while shaking at 200 rpm followed by heat inactivation. HindIII produces the 5' -TCGA- overhangs in the DNA which were filled with the nucleotides, one of which was biotin-14-dCTP (Invitrogen). At this stage, one tube for each sample was kept separately and used to prepare the 3C control libraries. To fill in and mark the DNA ends, 2 µl 10 mM dATP, 2 µl 10 mM dGTP, 2 µl 10 mM dTTP, 40 µl 0.4 mM biotin-14-dCTP and 10 µl 5U/µl Klenow (NEB) were added. The mixtures were incubated at 25°C for 30 minutes and subsequently placed on ice. Enzymes were inactivated by adding 100 µl 10% SDS to tubes and incubated at 65°C for 30 minutes and placed on ice immediately. The resulting blunt end fragments were ligated under a very dilute condition in order to favour the intra-molecular ligation events between the cross-linked fragments. For ligation each digested chromatin mixture were transferred to 15 ml tubes containing ligation mix [745 µl 10% Triton X-100, 745 µl 10x ligation buffer (500 mM Tris-HCl pH 7.5, 100 mM $MgCl_2$, 100 mM DTT), 80 µl 10 mg/ml BSA, 80 µl 100 mM ATP and 5.96 ml water] and transferred the digested chromatin into corresponding 15 ml tube. For 3C sample, 10U and 50U T4 DNA ligase for Hi-C sample were added to each tube and incubated at 16 °C for 4 hr. After ligation reverse crosslinking was performed by incubating the tubes at 65 °C for 16 hours after adding 30 µl 20 mg/ml proteinase K (Ambion). Additional 30 µl 20 mg/ml proteinase K was added next day and incubated for 1

hour. The reaction mixture was cooled to room temperature and transferred to 50 ml tube. The RNA contamination was removed by adding 10 µl of RNase A solution (Sigma R4642) and incubated at 37 °C for 30 minutes. DNA was extracted by adding an equal volume of phenol: chloroform: isoamyl alcohol (25:24:1 ratio) mixed thoroughly by vigorous shaking. The tubes were centrifuged for 10 minutes at 3,000g and transferred the aqueous phase to the new tubes and t this step was repeated again. An equal volume of sterile MilliQ, 1,500 µl of 3 M NaOAC (pH 5.6), 40 µl of glycogen (Ambion) and 25 ml of 100% ice-cold 100% ethanol was added and mix well by inverting the tube several times. Tubes were incubated at -80°C for at least two hours. The tubes were centrifuged at 4°C for 30 minutes at 10,000g. The supernatant was discarded and the pellet was washed with 70% ice-cold ethanol at 4°C for 10 minutes and pelleted again at 10,000g followed by air-drying. DNA pellet was dissolved in 500 µl, 10 mM TE (pH 7.5) and transferred to 1.5 ml tubes. Another round of DNA purification was performed similarly. Finally, the pellet was dissolved in 100 µl 10 mM TE (pH 7.5).

To prevent the pooling of non-ligated DNA fragment through Dynabead Myone streptavidin coated magnetic bead (Invitrogen) exonuclease activity of T4 DNA polymerase was used. T4 DNA polymerase removed the biotin-14-dCTP from non-ligated DNA end. Multiple 5 µg reactions for each Hi-C library was performed. For a unit of reaction 5 µg DNA, 1 µl 10 mg/ml BSA, 10 µl 10x NEBuffer 2, 4 µl 10 mM dNTP and 2 µl of 3U/µl T4 DNA polymerase (NEB) in a total volume of 100 µl was constituted and incubated at 12°C for 2 hours. Reactions were stopped by adding 2 µl 0.5 M EDTA pH 8.0 (Cat No. 03690 Sigma). The different reactions were pooled in sets in respective libraries to proceed for DNA purification. Phenol: chloroform (1:1) extraction was carried out followed by ethanol precipitation and the pellets were dissolved in 100 µl nuclease free water. The ligated DNA is then sheared with the covaris M220 focused-ultrasonicator (Covaris) with the setting, duty

factor 10%, peak incident power 50, cycle/burst 200, treatment times 60 second to get the fragment size of 300-600 base pairs. Sheared DNA was resolved on 1.5% agarose gel in 1X TAE and the desired fragment of size 300-600 was excised and eluted from the gel using gel extraction kit (Qiagen) according to the manufacturer. The DNA concentration was measured with the Quant-iT assay (Invitrogen). To minimize the non-specific binding Dynabead Myone C1 streptavidin beads were blocked with the I-block (Applied Biosystem) according to the John and Quin, 2008.

The biotin-containing fragments were pulled down with I-Block blocked Dynabead MyOne Streptavidin C1 Beads to remove non-biotinylated molecule to get libraries enriched with interacting fragments (biotinylated) according to the manufacturer. Once the DNA is bound to the beads, they are washed to remove non-specifically binding DNA. The Hi-C DNA (Covaris shearing generates dsDNA fragments with 3' or 5' overhangs.) ends were repaired on beads using End Repair Mix (Illumina) according to the manufacturer protocol. The 3' to 5' exonuclease activity of this mix removes the 3'overhangs and the polymerase activity fills in the 5' overhangs. A single ‘A’ nucleotide is added to the 3’ ends of the blunt fragments using A-Tailing Mix to prevent them from ligating to one another during the adapter ligation reaction. A corresponding single ‘T’ nucleotide on the 3’ end of the adapter provides a complementary overhang for efficient ligation of the adapter to the Hi-C DNA fragment. Ligation of the Illumina Paired-end Adapters was performed while the Hi-C DNA is bound to the streptavidin beads according to the manufacturer. Bead bounded DNA increases the efficiency of adapter ligation by decreasing the mobility of the DNA fragments and also facilitates removal of the unligated adaptor. After library preparation, Hi-C DNA fragments were selectively amplified those that have adaptor molecules on both ends using PCR. The PCR is performed with a PCR primer cocktail that anneals to the ends of the adapters according to the manufacturer (True Seq DNA LT kit, Illumina). The number of PCR cycles

should be minimized to avoid PCR artifacts. Primer dimers are removed from amplified DNA by using 1.8x AMpure XP bead (Beckman Coulter). Size and concentrations of purified libraries were quantified with Bioanalyzer and sequenced from both ends on HiSeq2500 platform (Illumina).

Additional References:

John, J. S., & Quinn, T. W. (2008). Rapid capture of DNA targets. Biotechniques, 44(2), 259-264.
