# Supplementary material for: Stress Conditions Modulate the Chromatin Interactions Network in Arabidopsis
Source: Front Genet. 2022 Jan 5;12:799805. doi: 10.3389/fgene.2021.799805 (PMC8766718; doi:10.3389/fgene.2021.799805)
Supplement: Supplementary file 4 [file DataSheet1.PDF]

Table S1: Identified significant interaction at 1Kb resolution and its classification based on the location of interacting region on chromosome.

Libraries	Total Interaction	Cis	Trans
Native Condition	3635	2698	937
High Temperature	5320	3731	1589
Salicylic Acid	3309	2288	1021
